# Complementary and Alternative Medicine for Idiopathic Parkinson’s Disease: An Evidence-Based Clinical Practice Guideline

**DOI:** 10.3389/fnagi.2018.00323

**Published:** 2018-10-15

**Authors:** Ki-Ho Cho, Tae-Hun Kim, Seungwon Kwon, Woo-Sang Jung, Sang-Kwan Moon, Chang-Nam Ko, Seung-Yeon Cho, Chan-Yong Jeon, Sang-Ho Lee, Tae Young Choi, Ji Hee Jun, Jiae Choi, Myeong Soo Lee, Eun Kyoung Chung

**Affiliations:** ^1^Department of Cardiology and Neurology, College of Korean Medicine, Kyung Hee University, Seoul, South Korea; ^2^Korean Medicine Clinical Trial Center, Korean Medicine Hospital, Kyung Hee University, Seoul, South Korea; ^3^Stroke and Neurological Disorders Center, Kyung Hee University Hospital at Gangdong, Seoul, South Korea; ^4^Department of Korean Internal Medicine, College of Korean Medicine, Gachon University, Seongnam, South Korea; ^5^Gangdong Mokhuri Oriental Medical Hospital, Seoul, South Korea; ^6^Clinical Medicine Division, Korea Institute of Oriental Medicine, Daejeon, South Korea; ^7^Integrative Health Promotion Team, Korea Health Promotion Institute, Seoul, South Korea; ^8^Division of Clinical Pharmacy, Department of Pharmacy, College of Pharmacy, Kyung Hee University, Seoul, South Korea

**Keywords:** idiopathic Parkinson’s disease, complementary and alternative medicine, evidence-based medicine, clinical practice guideline, recommendations

## Abstract

Patients with idiopathic Parkinson’s disease (IPD) require long-term care and are reported to use complementary and alternative medicine (CAM) interventions frequently. This CAM-specific clinical practice guideline (CPG) makes recommendations for the use of CAM, including herbal medicines, acupuncture, moxibustion, pharmaco-acupuncture, and qigong (with Tai chi) in patients with IPD. This guideline was developed using an evidence-based approach with randomized controlled trials currently available. Even though this CPG had some limitations, mainly originating from the bias inherent in the research on which it is based, it would be helpful when assessing the value of the CAM interventions frequently used in patients with IPD.

## Introduction

Idiopathic Parkinson’s disease is a chronic neurodegenerative disease which presents progressive loss of dopaminergic neurons in substantia nigra. Generally, long-term medical care is often required after first diagnosis of IPD. The prevalence of IPD rises with age but there could be geographic difference in the prevalence among 70–79-year-old population between Asian countries and North America–Europe countries (Pringsheim et al., 2014). Because disease modifying therapy for IPD is absent and patients with IPD have unmet needs which are introduced from undesirable symptoms related to the conventional levodopa therapy and various non-motor symptoms (Bastide et al., 2015), many IPD patients use various complementary and alternative therapies currently.It has been reported that 25.6–76% of patients with IPD have experience of CAM treatment (Wang et al., 2013). Although types of interventions that are frequently used were reported to be a little bit different between in Asia (acupuncture and herbal medication are most frequently used two interventions) and in United States (dietary supplements and massage are most frequently used two interventions) (Wang et al., 2013), many CAM interventions have been shown to be effective in motor function and balancing of IPD patients in clinical studies (Lee et al., 2008; Kim et al., 2012; Yang et al., 2014). However, there is no specific CPG currently available for CAM interventions in patients with the disease. Several interventions, including acupuncture, biofeedback, and manual therapy, were mentioned in part of a CPG endorsed by the Canadian Neurological Sciences Federation and Parkinson Society Canada in 2011, but with the caveat that the evidence for their use was insufficient and in need of update (Grimes et al., 2012). Another CPG for integrative interventions published in 2015 included a comprehensive CAM intervention-specific CPG for IPD but was a consensus-based guideline that was not developed on the basis of the current best clinical evidence (Pan et al., 2015). An up-to-date, evidence-based, specific CPG on CAM interventions for IPD is needed to provide clinical evidence that can assist both health care professionals and patients when making a decision whether to include CAM interventions in their long-term strategy for management of the disease.

This CAM-specific CPG for IPD was developed to provide reliable recommendations for health care professionals and patients based on current best clinical evidence on the benefits and harms of CAM interventions.

The scope covered in this guideline contained any modalities which are originated from outside of conventional biomedicine and can be used together with main-stream medical practice (Clarke et al., 2015). In the course of developing this guideline, we aimed to suggest recommendations the interventions that are commonly used in Korea in the highest priority, considering the reality of Korea. We plan to expand the scope of complementary medicine in future revisions to cover various interventions not covered in this guideline.

## Materials and Methods

**FIGURE 1:**
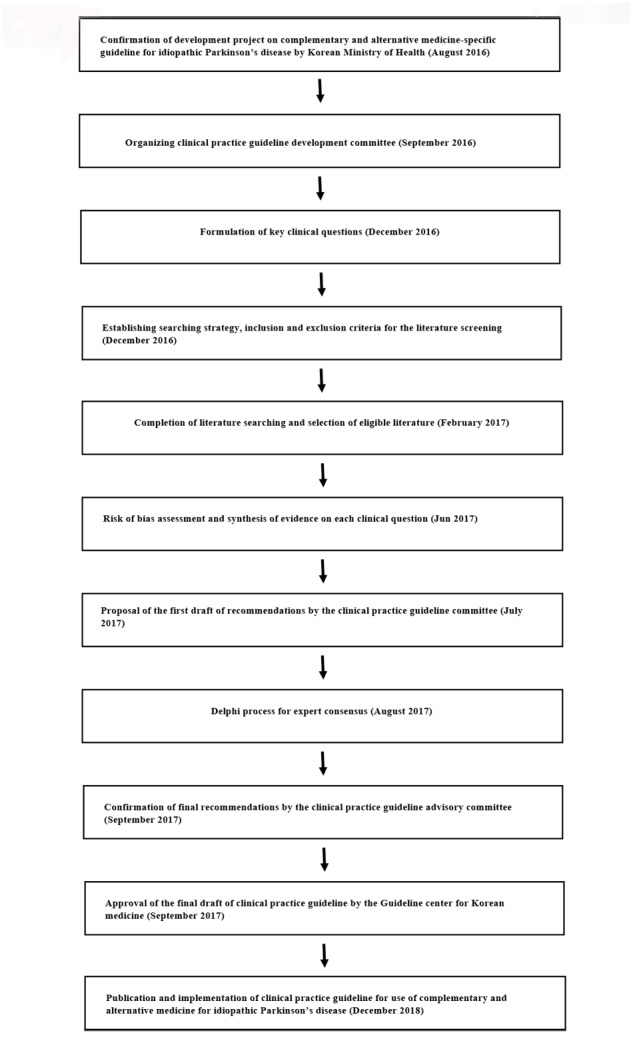
The clinical practice guideline development process.

This CAM-specific CPG for IPD was developed by the Society of Stroke on KM (**Figure 1**). In September 2016, the society organized a steering committee to oversee the development of the guideline, a development committee to synthesize the evidence and draft the CPG, and an advisory committee to review the proposed CPG. The steering committee included six society members, the development committee was multidisciplinary and consisted of four disease specialists, five methodology experts, one statistician, and one economic analysis specialist. The advisory committee included seven KM physicians working in primary and secondary care and recommended by the Society of Stroke on KM and two KM physicians recommended by the panel of physicians at the Guideline center for KM.

The development committee identified the primary clinical questions involving herbal medicines, acupuncture, moxibustion, qigong, and Tai chi which were reported to be most common and clinically important CAM interventions for IPD patients (Kim et al., 2009; Wang et al., 2013) and developed a search strategy and inclusion/exclusion criteria for application to the relevant literature. The PubMed, Cochrane CENTRAL, EMBASE, CNKI, Oriental Medicine Advanced Searching Integrated System, and National Digital Science Library databases were searched up to February 2017 to identify eligible RCTs. The details of the search strategy used for each intervention is shown in **Supplement 1**.

Multiple members of the development committee selected the literature, extracted the relevant data, and performed the risk of bias assessment and meta-analysis. Any disagreement was resolved by group discussion. The evidence for each clinical question was qualitatively and quantitively synthesized. In case of multiple clinical trials using the same specific intervention such as specific type of herbal medications or acupuncture, clinical questions for specific interventions were generated through panel discussion and synthesized too. Levels of evidence and the strength of the recommendations were then evaluated using the GRADE approach (Guyatt et al., 2008). Validated, Parkinson’s disease-specific outcomes including the UPDRS, levodopa dose, 39-Item Parkinson’s disease questionnaire (PDQ-39), Total effectiveness rate, Webster scale score, Berg balance scale, Parkinson Disease Sleep Scale, and adverse events were assessed for GRADE. The level of evidence for each outcome was classified as high, moderate, low, or insufficient based on the overall risk of bias, inconsistency, indirectness, imprecision, and other considerations in the included studies as suggested by the GRADE working group (**Table 1**). The strength of the recommendations was graded according to the level of evidence and its clinical importance, such as the risk and benefit of the intervention and its value to the patient, as A, B, C, D, or GPP (**Table 2**). Based on the level of evidence and strength of the recommendations, a draft recommendation for each clinical question was prepared by the development committee.

**Table 1:** Levels of evidence.

Level	Description
High	We are very confident that the true effects lie close to that of the estimate of effect.
Moderate	We are moderately confident in the effect estimate: the true effect is likely to be close to the estimate of effect, but there is a possibility that it is substantially different.
Low	Our confidence in the effect estimate is limited: The true effect may be substantially different from the estimate of the effect.
Insufficient	We have very little confidence in the effect estimate: The true effect is likely to be substantially different from the estimate of effect.


**Table 2:** Grade of recommendation.

Grade	Definition	Notation
A	Recommended when the level of evidence is “high,” there is a clear benefit and the level of use in clinical settings is high.	Is recommended
B	Assigned when the level of evidence is “moderate,” the benefits are reliable, and the level of use in clinical settings is high or moderate. Although relevant studies providing evidence supporting the recommendation may be lacking, the clinical benefit is clear.	Should be considered
C	Assigned when the level of evidence is “low,” the benefits are not reliable, but the recommendation’s level of use in clinical settings is high or moderate.	May be considered
D	Assigned when the level of evidence is “low” or “insufficient,” the benefits are unreliable, harmful results may arise, and the recommendation’s level of use in clinical settings is low.	Is not recommended
GPP	Due to the lack of evidence-based medical information, the level of evidence is “low” or “insufficient” and the benefits cannot be evaluated. This rating is assigned based on the clinical experience of the group that developed the CPG and a high level of use in clinical settings.	Is recommended based on the clinical experience of the group that developed the CPG


*CPG, clinical practice guideline; GPP, good practice point.*

The Delphi method was used to achieve formal consensus between experts and clinicians and required all contributors to complete an on-line web survey^1^. A questionnaire concerning the initial draft of the recommendations was prepared, and a panel of nine multidisciplinary experts was established on the recommendation of the advisory committee. The experts on the panel were asked to grade their degree of agreement with each recommendation on a nine-point Likert scale (1, totally disagree; 9, fully agree). A median value of ≥7 indicated that agreement was reached and a median value <7 implied disagreement that required a further round of the Delphi process. The expert panel answered two rounds of questionnaires, after which the final recommendation for the CPG was drafted (**Supplementary Table 1**). The Guideline center for KM then approved the final draft of the CPG to be implemented by the end of 2018.

## Results

### Herbal Medicine

#### Clinical Question 1

##### Is concomitant administration of herbal medicines and anti-parkinsonian drug therapy a more effective symptomatic treatment for IPD than monotherapy with anti-parkinsonian agents?

This clinical question was intended to assess the overall clinical evidence for combination therapy using herbal medicines of any type and anti-parkinsonian agents. Although inclusion of multiple prescriptions when assessing the clinical evidence for herbal medicine is controversial, it is considered necessary at the present stage. In addition, the benefits and risks of treatment are similar between the herbal medicines available, and there was a consensus among the group of experts that the values and preferences for these medicines are similar. After assessing the overall evidence for herbal medicine regardless of the type of prescription, we evaluated the clinical evidence for several herbal prescriptions that have been evaluated in previous clinical trials using the following clinical questions in below sections.

Seventy-five studies (including 5430 patients with IPD) that evaluated the UPDRS total score were included. The score was significantly lower in the combination therapy group than in the anti-parkinsonian monotherapy group (MD -6.06, 95% CI [-6.82, -5.3]). The results of 44 RCTs (including 3100 patients with IPD) that evaluated the UPDRS part I score for mentation, behavior, and mood favored combination therapy (MD -1.6, 95% CI [-1.94, -1.26]). Fifty-nine RCTs (including 4311 patients with IPD) that evaluated the UPDRS part II score for activities of daily living found a significant difference between the combination therapy group and the anti-parkinsonian drug monotherapy group (MD -2.2, 95% CI [-2.67, -1.76]). Seventy RCTs (including 4909 patients with IPD) that evaluated the UPDRS part III score for motor examination were included, and combination treatment achieved better results (MD -3.41, 95% CI [-4.23, -2.59]). When the UPDRS part IV score for motor complications was assessed, the results of 40 studies (including 3078 patients with IPD) indicated a significant difference in favor of combination treatment (MD -1.41, 95% CI [-1.72, -1.10]). The PDQ-39 summary index was assessed in 20 studies (including 1490 patients with IPD). The MD in the summary effect estimate for the PDQ-39 summary score was 9.29 (95% CI [-10.83, -7.75]) in favor of combination therapy. Twenty-seven RCTs (including 2043 patients with IPD) included information on the concomitant dose of levodopa. The SMD in levodopa consumption was -0.77 (95% CI [-0.99, -0.56]) when the group that received combination therapy was compared with the group that received anti-parkinsonian drug therapy alone, suggesting that lowering the dose of levodopa in the combination group was more effective. Forty-nine RCTs (including 3463 patients with IPD) included an assessment of adverse events; the RR for total incidence was estimated to be 0.44 (95% CI [0.3, 0.52]) in the group that received combination therapy when compared with the group that received anti-parkinsonian drug therapy alone. However, the overall level of evidence for these analyses was assessed as low, and the results of future studies will have a significant impact on the degree of confidence in the summary effect estimates. A combination of herbal medicine and anti-parkinsonian drug therapy should be considered in patients with IPD; however, the strength of this recommendation may change according to the outcomes of future research (strength of recommendation, B). The evidence in this section suggests a comprehensive recommendation for combination therapy using various herbal medicines and anti-parkinsonian drugs (**Supplementary Table 2.1**).

#### Recommendation

Concomitant treatment with herbal medicines and anti-parkinsonian drugs should be considered in patients with IPD (strength of recommendation: B/level of evidence: low).

#### Clinical Question 1-1

##### Does administration of Bosin-yanggan-sigpung-bang herbal medicine with anti-parkinsonian drug therapy improve symptoms more than anti-parkinsonian drugs alone in patients diagnosed with IPD?

Bosin-yanggan-sigpung-bang has been one of the most frequently evaluated herbal medicines in clinical trials and consists mainly of *Rehmannia glutinosa*, *Paeonia lactiflora*, and *Polygonum multiflorum*. Four RCTs (including 289 patients with IPD) that compared Bosin-yanggan-sigpung-bang and levodopa combination therapy and levodopa monotherapy were included. The MD in the summary effect estimate for the UPDRS total score was -11.39 (95% CI [-16.2, -6.57]) and the SMD for consumption of levodopa was -1.04 (95% CI [-1.49, -0.58]), which suggests a statistically significant difference in favor of combination treatment. There was no significant difference in the incidence of adverse events between the two groups. The levels of evidence for the UPDRS total score and the dose of levodopa were inadequate (**Supplementary Table 2.2**).

#### Recommendation

Concomitant administration of Bosin-yanggan-sigpung-bang herbal medicine and anti-parkinsonian drug therapy may be considered in patients with IPD (strength of recommendation: C/level of evidence: insufficient).

#### Clinical Question 1-2

##### Does administration of Bosin-hwalhyeol-cheobang herbal medicine with anti-parkinsonian drug therapy improve symptoms more than anti-parkinsonian drug therapy alone in patients diagnosed with IPD?

Bosin-hwalhyeol-cheobang contains mainly *Cornus officinalis*, *P. multiflorum*, and *Salvia miltiorrhiza*. Ten studies that were included in the analysis were used to answer this clinical question. The summary effect estimates for the UPDRS total score (MD -6.32, 95% CI [-8.6, -4.05]) and PDQ-39 (MD -9.01, 95% CI [-2.52, 0.25]) were in favor of combination therapy. The RR for the overall incidence of adverse events was 0.46 (95% CI [0.21, 1.03]; **Supplementary Table 2.3**).

#### Recommendation

Concomitant use of Bosin-hwalhyeol-cheobang herbal medicine and anti-parkinsonian drug therapy may be considered in patients with IPD (strength of recommendation: C/level of evidence: moderate).

#### Clinical Question 1-3

##### Does administration of Bosin-hwalhyeol-tonglag-cheobang herbal medicine with anti-parkinsonian drug therapy improve symptoms more than anti-parkinsonian drug therapy alone in patients diagnosed with IPD?

Bosin-hwalhyeol-tonglag-cheobang is mainly composed of *Lycium chinense*, *C. officinalis*, *P. multiflorum*, *S. miltiorrhiza*, and *Buthus martensi*. Three studies could be included in the analysis. When comparing combination therapy and levodopa monotherapy, the MD for the UPDRS total score was -15.40 (95% CI [-19.80, -11.00]) and the MD for the PDQ-39 was -7.05 (95% CI [-11.80, -2.30]) in favor of combination therapy. The overall level of evidence was moderate. None of the studies included information on adverse events (**Supplementary Table 2.4**).

#### Recommendation

Concomitant administration of Bosin-hwalhyeol-tonglag-cheobang herbal medicine and anti-parkinsonian drug therapy may be considered in patients with IPD (strength of recommendation: C/level of evidence: moderate).

#### Clinical Question 1-4

##### Does administration of Sugji-pyeongjeon-tang herbal medicine with anti-parkinsonian drugs improve symptoms more than anti-parkinsonian drug therapy alone in patients diagnosed with IPD?

Sugji-pyeongjeon-tang is a herbal prescription that consists mainly of *R. glutinosa*, *L. chinense*, *Loranthus parasiticus*, and *Gastrodia elata*. Three studies could be included in the analysis. The summary effect estimates for the UPDRS part II score for activities of daily living (MD -1.59, 95% CI [-2.51, -0.67]) and those for the UPDRS part III score for motor examination (MD -2.51, 95% CI [-3.89, -1.13]) favored combination therapy. The MD in the summary effect estimates for the amount of levodopa used was 0.05 (95% CI [-0.26, 0.36]). The estimated incidence of total adverse events favored combination treatment (RR 0.80, 95% CI [0.25, 2.52]). The level of evidence was low (**Supplementary Table 2.5**).

#### Recommendation

Concomitant use of Sugji-pyeongjeon-tang herbal medicine and anti-parkinsonian drug therapy should be considered in patients with IPD (strength of recommendation: B/level of evidence: low).

### Acupuncture

#### Clinical Question 2

##### Does a combination of anti-parkinsonian drug therapy and acupuncture improve symptoms more than anti-parkinsonian drug therapy alone in patients diagnosed with IPD?

This clinical question was intended to assess the overall clinical evidence for combination therapy with acupuncture and anti-parkinsonian medications regardless of type of acupuncture intervention. After addressing this clinical question, we assessed the clinical evidence for the frequently used acupuncture interventions in the following clinical questions. Twenty-one RCTs were included. A meta-analysis of clinical trials comparing combination treatment with acupuncture and anti-parkinsonian drug therapy with anti-parkinsonian drug therapy alone suggested that combination therapy had better overall clinical effectiveness than anti-parkinsonian drug therapy alone (RR 1.2, 95% CI [1.08, 1.33]). In addition, there was a statistically significant difference in the Webster scale score (MD -3.09, 95% CI [-4.8, -1.38]) in favor of combination treatment. The UPDRS total score suggested that combination therapy with acupuncture and anti-parkinsonian drug administration was more effective in improving symptoms than anti-parkinsonian drug therapy alone (MD -6.72, 95% CI [-10.24, -3.2]). Only two studies reported adverse events, with no serious adverse events occurring in either study. The overall level of evidence is low, and future studies will have a significant impact on the degree of confidence in the summary effect estimates. This section suggests a comprehensive recommendation for various acupuncture interventions, and recommendations for individual type of acupuncture are suggested below (**Supplementary Table 3.1**).

#### Recommendations

Concomitant use of acupuncture and anti-parkinsonian drug therapy should be considered in patients with IPD (strength of recommendation: B/level of evidence: low).

Acupuncture points such as GB20, LR3, GB34, LI4, GV20, KI39, LI11, GV16, BL10, BL40, GB6, GV1, and PC6 can be considered for use in acupuncture.

#### Clinical Question 2-1

##### Does combination treatment with anti-parkinsonian drug therapy and manual acupuncture improve symptoms more than anti-parkinsonian drug therapy alone in patients diagnosed with IPD?

Seven RCTs were available to address this clinical question. The anti-parkinsonian therapy used in the control groups was a fixed combination of levodopa and benserazide in all cases. Significant improvement in the Webster scale score (MD -3.75, 95% CI [-5.27, -2.23]) was observed in favor of combination therapy when compared with the fixed combination as monotherapy. Adverse events were only reported in two of the seven studies. There were no serious adverse events in either of these studies. The level of evidence from the literature is low and the results of future studies are likely to have a significant impact on the degree of confidence in the summary effect estimates (**Supplementary Table 3.2**).

#### Recommendation

Concomitant use of manual acupuncture and anti-parkinsonian drug therapy should be considered in patients with IPD (strength of recommendation: B/level of evidence: low).

#### Clinical Question 2-2

##### Does combination treatment with anti-parkinsonian drug therapy and electroacupuncture improve symptoms more than anti-parkinsonian drug therapy alone in patients diagnosed with IPD?

In three RCTs, electroacupuncture and anti-parkinsonian drugs were compared with anti-parkinsonian drug therapy alone and there was no significant difference in overall clinical effectiveness (RR 1.22, 95% CI [0.91, 1.63]), Webster scale score (MD -2.98, 95% CI [-6.11, 0.15]), or UPDRS total score (MD -3.8, 95% CI [-7.74, 0.15]) between the two groups. However, the level of evidence was low and the results of future studies are likely to have a significant impact on the degree of confidence in the summary effect estimates (**Supplementary Table 3.3**).

#### Recommendation

Concomitant use of electroacupuncture and anti-parkinsonian drug therapy may be considered in patients with IPD (strength of recommendation: C/level of evidence: low).

If the patient’s chief complaints are tremor and rigidity, electroacupuncture should be applied carefully so as not to trigger overstimulation.

#### Clinical Question 2-3

##### Does a combination of anti-parkinsonian drug therapy and scalp acupuncture improve symptoms more that anti-parkinsonian drug therapy alone in patients with IPD?

Three RCTs were available to answer this clinical question. The fixed combination of levodopa and benserazide was used as the anti-parkinsonian drug therapy in all the control groups. There was significant improvement in the Webster scale score (MD -1.97, 95% CI [-3.73, -0.21) in favor of combination therapy. There was no description of the adverse events that occurred in any of the studies. The level of evidence in the literature is insufficient but combination treatment is recommended based on the clinical experience of the development committee (**Supplementary Table 3.4**).

#### Recommendation

Concomitant use of scale acupuncture and anti-parkinsonian drugs may be considered in patients with IPD (strength of recommendation: GPP/level of evidence: insufficient).

### Moxibustion

#### Clinical Question 3

##### Does moxibustion improve symptoms in patients with IPD?

Five studies (including 293 patients with IPD) were included in the comparison of the effectiveness of moxibustion with that of other interventions in terms of the UPDRS total score. The UPDRS total score was lower in the moxibustion group than in the control group (MD -8.75, 95% CI [-12.54, -4.95]). Three RCTs (including 174 patients with IPD) were included in the assessment of the UPDRS part III score for motor examination, and a significant difference was observed in favor of moxibustion (MD -1.92, 95% CI [-3.00, -0.84]). Only one study reported adverse events and none of the studies reported side effects. The overall level of evidence was low. Although moxibustion could potentially be a treatment for patients with IPD, there is a lack of concrete evidence for its efficacy and safety (**Supplementary Table 4.1**).

#### Recommendation

Moxibustion may be considered in patients with IPD (strength of recommendation: C/level of evidence: low).

Care should be taken not to cause adverse events by excessive stimulation if the main symptoms are tremor and rigidity.

#### Clinical Question 3-1

##### Does combination treatment with direct moxibustion, acupuncture, and anti-parkinsonian drug therapy improve symptoms in patients diagnosed with IPD?

Only one RCT could be included in the analysis to answer this clinical question. Sixty patients with IPD were included in this study and the UPDRS total score was assessed by comparing the combination of direct moxibustion, acupuncture, and anti-parkinsonian drug therapy with anti-parkinsonian drug monotherapy alone. The UPDRS total score was significantly improved in the combination group in comparison with the anti-parkinsonian drug monotherapy group (MD -7.07, 95% CI [-11.30, -2.84]). There was no description of adverse events in this study. The level of evidence is low; however, summary effect estimates may change according to the findings of future research. Care should be taken to avoid adverse events by excessive stimulation if the patient’s main symptoms are tremor and rigidity (**Supplementary Table 4.2**).

#### Recommendation

A combination of direct moxibustion, acupuncture, and anti-parkinsonian drug therapy may be considered in patients with IPD (strength of recommendation: C/level of evidence: low). Care should be taken not to cause adverse events by excessive stimulation if the patient’s main symptoms are tremor and rigidity.

#### Clinical Question 3-2

##### Does a combination of Moxa-stick moxibustion and anti-parkinsonian drug therapy improve symptom in patients diagnosed with IPD?

One RCT would be included in the analysis to answer this clinical question. There was no significant difference in the UPDRS total score between combination therapy and a fixed combination of levodopa and benserazide (MD -5.48, 95% CI [-11.64, 0.68]). No information on adverse events was available. The overall level of evidence is low. Care should be taken not to cause adverse events by excessive stimulation if the patient’s main symptoms are tremor and rigidity (**Supplementary Table 4.3**).

#### Recommendation

Concomitant treatment with Moxa-stick moxibustion and anti-parkinsonian drug therapy may be considered in patients with IPD (strength of recommendation: C/level of evidence: low).

Care should be taken not to cause adverse events by excessive stimulation if the patient’s main symptoms are tremor and rigidity.

#### Clinical Question 3-3

##### Does combination treatment with warm-needling acupuncture and swallowing exercises improve dysphagia symptoms in patients diagnosed with IPD?

One RCT comparing a combination of warm-needling acupuncture and swallowing exercises with swallowing exercises alone was included in the analysis to address this clinical question. The total effectiveness rate was significantly higher in the combination treatment group (RR 1.67, 95% CI [1.11, 2.50]) but there was no report on adverse events. The overall level of evidence was low (**Supplementary Table 4.4**).

#### Recommendation

Combination treatment with warm-needling acupuncture and swallowing exercises may be considered for the treatment of dysphagia in patients with IPD (strength of recommendation: C/level of evidence: low).

### Pharmaco-acupuncture

#### Clinical Question 4-1

##### Does a combination of bee venom acupuncture and anti-parkinsonian drug therapy improve symptoms in patients diagnosed with IPD?

One RCT was available to address this clinical question. When comparing a combination of bee venom acupuncture and anti-parkinsonian drug therapy with anti-parkinsonian drug therapy alone, there was a significant difference in UPDRS total score in favor of combination therapy. One participant reported an itching event after bee venom acupuncture. The overall level of evidence was insufficient, but the development committee decided to make a GPP recommendation based on the prevalent use of this treatment modality in current clinical practice and the opinion of the expert committee.

#### Recommendation

Concomitant treatment with bee venom acupuncture and anti-parkinsonian drug therapy is recommended in patients with IPD (strength of recommendation: GPP/level of evidence: insufficient).

#### Clinical Question 4-2

##### Does a combination of pharmaco-acupuncture and anti-parkinsonian drug therapy improve symptoms in patients diagnosed with IPD?

One RCT could be included in the analysis to answer this clinical question. There was no significant difference in the total effectiveness rate between a combination comprising Puerarin pharmaco-acupuncture and anti-parkinsonian therapy and single anti-parkinsonian drug therapy alone (RR 1.06, 95% CI [0.90, 1.26]). No adverse events were reported. The overall level of evidence was insufficient, but the development committee decided to make a GPP recommendation based on the prevalent use in current clinical practice and the opinion of the expert committee (**Supplementary Table 5**).

#### Recommendation

Concomitant treatment with pharmaco-acupuncture and anti-parkinsonian drug therapy is recommended in patients with IPD (strength of evidence: GPP/level of evidence: insufficient).

### Qigong and Tai Chi

#### Clinical Question 5-1

##### Does a combination of Qigong, walking exercise, and anti-parkinsonian drug therapy improve motor function and sleep quality in patients diagnosed with IPD?

Two RCTs (including 141 patients with IPD) were included to answer this clinical question. The group of patients that received combination therapy (qigong, walking exercise, anti-parkinsonian drugs) had a better UPDRS part III score for motor examination (MD -4.17, 95% CI [-5.43, -2.92]) and a better Berg balance scale score (MD 3.30, 95% CI [2.62, 3.98]). In addition, the combination therapy group showed better results in several domains on the Parkinson Disease Sleep Scale-2, including sleep quality (MD -11.47, 95% CI [-15.77, -7.17]), motor symptoms at night (MD -4.63, 95% CI [-6.02, -3.24]), symptoms of Parkinson’s disease at night (MD -3.1, 95% CI [-4.37, -1.83]), and disturbed sleep (MD -3.44, 95% CI [-5.09, -1.79]) compared with the groups without qigong. However, the overall level of evidence was low (**Supplementary Table 6.1**).

#### Recommendation

Concomitant treatment with qigong, walking exercise, and anti-parkinsonian drug therapy should be considered in patients with IPD (strength of recommendation: B/level of evidence: low).

#### Clinical Question 5-2

##### Does Tai chi improve motor function in patients diagnosed with IPD?

Three RCTs (including 216 patients with IPD) were included for evaluation of this clinical question. When compared with exercise or stretching movement, Tai chi had a better effect on the UPDRS part III score for motor examination (MD -3.1, 95% CI [-3.86, -2.34]) and Berg balance scale score (MD 3.52, 95% CI [1.92, 5.12]). In addition, there was a significant decrease in the number of falls recorded in the Tai chi training group (OR 0.38, 95% CI [0.19, 5.12]). However, the overall level of evidence was low (**Supplementary Table 6.2**).

#### Recommendations

Tai chi can be considered in patients with IPD (strength of recommendation: B/level of evidence: low).

## Discussion

This evidence-based CPG on CAM interventions for IPD outlines the currently available evidence and makes recommendations for the commonly used CAM interventions, i.e., herbal medicines, acupuncture, moxibustion, pharmaco-acupuncture, and qigong (with Tai chi). This CPG mainly addresses combination treatment using individual CAM interventions and anti-parkinsonian drug therapy because of the current lack of clinical evidence for use of CAM interventions alone. In this sense, CAM interventions need to be considered for concomitant use only until new and conclusive evidence of their efficacy as monotherapy emerges. In addition to this, about CAM interventions usage, communication between physician and patients is very important to prevent potential adverse events, which should be reminded when CAM interventions get started (Wang et al., 2013).

### Limitations

The development committee acknowledges that this CPG has several limitations, the most important one being that the overall level of evidence for each intervention as assessed by GRADE approach is low or insufficient because of the high risk of bias introduced by methodologic problems and inaccuracies arising from the small numbers of participants in the included studies. Indeed, there could be considerable publication bias because most of the relevant studies were performed in Asian countries. Additionally, we did not intend to limit target population of this CPG but most clinical studies included only Asian population who showed different characteristics in ages, sex, and region across the studies. Second, most of the recommendations focus on the overall benefit and harm of combination treatment with CAM interventions and antiparkinsonian drugs. CPGs that focus on specific symptoms or long-term complications related to IPD need to be developed in the future. In addition, this CPG dose not include detailed information on the methods for usage of individual CAM interventions, which would be updated in the future CPG. Third, this CPG is based on evidence for combination treatment with CAM interventions and anti-parkinsonian drug therapy. As already mentioned, there is no clear evidence regarding whether individual CAM interventions are effective and safe enough to be recommended in this CPG. Therefore, in principle, we recommend that most CAM interventions be used in combination with conventional anti-parkinsonian drug therapy at this stage. Third, there was no participation of consumer groups in the Delphi process, which means that no patient perspectives or preferences were taken into account in the development of this CPG. Fourth, no cost or economic analysis was performed. Finally, only limited interventions were assessed in this review. As we described in the “Materials and Methods” section, interventions which were frequently used in Asian countries were included for this CPG. Actually, frequently used CAM modalities are reported to be different between countries (Wang et al., 2013) so other interventions such as natural products, mind–body interventions, or Ayurveda should be assessed. In the future updated CPG, we will include these frequently used interventions which was not discussed in the CPG.

### Implications for Clinical Studies and Updates to the CPG

Rigorous RCTs for individual CAM interventions with adequate statistical power should be conducted, especially in the United States and Europe. The safety and effectiveness of CAM as monotherapy and when used as part of combination therapy should be evaluated in the IPD population. Comparative ranking between included CAM modalities would be helpful which can be suggested through indirect comparison using network meta-analysis method. A consumer group should be included in the Delphi process when updating this CPG in the future. Inclusion of a cost-effectiveness analysis should also be considered when updating this CPG. Finally, when clinically implementing recommendations of the interventions included in this CPG outside Korea, physicians and patients need to consider accessibility and feasibility of the interventions in their medical environment and context.

Development of this guideline was funded by the Ministry of Health & Welfare through the Korea Health Industry Development Institute. None of the members of the Society of Stroke on Korean Medicine or members of each committee and expert panel that participated in the Delphi process has any conflicts of interests in this research.

This guideline will be updated in 2020. In the future update, various CAM interventions not covered in this guideline will be included.

## Author Contributions

K-HC, T-HK, SK, W-SJ, S-KM, C-NK, S-YC, C-YJ, S-HL, TC, JJ, JC, ML, and EC participated in the CPG development committee and devised the CPG development project. K-HC, T-HK, SK, W-SJ, S-KM, C-NK, S-YC, C-YJ, and S-HL involved in the clinical question development. TC, JJ, JC, and ML conducted systematic literature searching and conducted electronic Delphi surveys. T-HK, TC, JJ, and JC conducted evidence synthesis and made draft of the recommendations. T-HK drafted this manuscript.

## Conflict of Interest Statement

The authors declare that the research was conducted in the absence of any commercial or financial relationships that could be construed as a potential conflict of interest.

## Footnotes

1 https://ko.surveymonkey.com

## Abbreviations

CAM: complementary and alternative medicine
CI: confidence interval
CPG: clinical practice guideline
GPP: good practice point
GRADE: Grading of Recommendation Assessment, Development, and Evaluation
IPD: idiopathic Parkinson’s disease
KM: Korean medicine
MD: mean difference
OR: odds ratio
RCT: randomized controlled trials
RR: relative risk
SMD: standardized mean difference
UPDRS: Unified Parkinson’s Disease Rating Scale
