# Supplementary material for: Complementary and Alternative Medicine for Idiopathic Parkinson’s Disease: An Evidence-Based Clinical Practice Guideline
Source: Front Aging Neurosci. 2018 Oct 15;10:323. doi: 10.3389/fnagi.2018.00323 (PMC6196228; doi:10.3389/fnagi.2018.00323)
Supplement: Supplementary file 1 [file Table_1.docx]

Supplement 1: Searching strategies and the Preferred Reporting Items for Systematic Reviews and Meta-Analyses (PRISMA) flow diagram for each clinical question

1. Searching strategy for herbal medicine

(1) [CENTRAL]

| #1 | MeSH descriptor: [Parkinson Disease] explode all trees |
| --- | --- |
| #2 | Parkinson*:ti,ab,kw (Word variations have been searched) |
| #3 | #1 or #2 |
| #4 | MeSH descriptor: [Herbal Medicine] explode all trees |
| #5 | MeSH descriptor: [Medicine, Traditional] explode all trees |
| #6 | MeSH descriptor: [Phytotherapy] explode all trees |
| #7 | MeSH descriptor: [Plants, Medicinal] explode all trees |
| #8 | MeSH descriptor: [Plant Preparations] explode all trees |
| #9 | MeSH descriptor: [Drugs, Chinese Herbal] explode all trees |
| #10 | herb* or plant* or phytodrug* or decoction*:ti,ab,kw (Word variations have been searched) |
| #11 | MeSH descriptor: [Medicine, Chinese Traditional] explode all trees |
| #12 | MeSH descriptor: [Medicine, Korean Traditional] explode all trees |
| #13 | "tokishakuyakusan":ti,ab,kw (Word variations have been searched) |
| #14 | "hachimijiogan":ti,ab,kw (Word variations have been searched) |
| #15 | "goshajinkigan":ti,ab,kw (Word variations have been searched) |
| #16 | rikkunshito:ti,ab,kw (Word variations have been searched) |
| #17 | "saikokeishito":ti,ab,kw (Word variations have been searched) |
| #18 | "hochuekkito":ti,ab,kw (Word variations have been searched) |
| #19 | "shakuyakukanzoto":ti,ab,kw (Word variations have been searched) |
| #20 | yokukansan:ti,ab,kw (Word variations have been searched) |
| #21 | "kamishoyosan":ti,ab,kw (Word variations have been searched) |
| #22 | #4 or #5 or #6 or #7 or #8 or #9 or #10 or #11 or #12 or #13 or #14 or #15 or #16 or #17 or #18 or #19 or #20 or #21 |
| #23 | #13 and #38 |

(2) [EMBASE]

| #1 | 'parkinson disease'/exp |
| --- | --- |
| #2 | parkinson*:ab,ti |
| #3 | 'parkinson disease'/exp OR parkinson*:ab,ti |
| #4 | 'herbal medicine'/exp OR 'traditional medicine'/exp OR 'phytotherapy'/exp OR 'medicinal plant'/exp OR 'plant medicinal product'/exp OR 'korean medicine'/exp OR 'herbaceous agent'/exp |
| #5 | tokishakuyakusan:ti,ab OR 'hachimijiogan':ti,ab OR 'goshajinkigan':ti,ab OR 'rikkunshito':ti,ab OR 'saikokeishito':ti,ab OR 'hochuekkito':ti,ab OR 'shakuyakukanzoto':ti,ab OR 'yokukansan':ti,ab OR 'kamishoyosan':ti,ab |
| #6 | herb*:ti,ab OR plant*:ti,ab OR phytodrug*:ti,ab OR decoction*:ti,ab OR botanical:ti,ab OR phytomedicine:ti,ab |
| #7 | chinese AND adj3 AND (medic*:ti,ab OR herb*:ti,ab OR drug*:ti,ab OR formul*:ti,ab OR plant*:ti,ab OR prescri*:ti,ab) |
| #8 | ('herbal medicine'/exp OR 'traditional medicine'/exp OR 'phytotherapy'/exp OR 'medicinal plant'/exp OR 'plant medicinal product'/exp OR 'korean medicine'/exp OR 'herbaceous agent'/exp) OR (tokishakuyakusan:ti,ab OR 'hachimijiogan':ti,ab OR 'goshajinkigan':ti,ab OR 'rikkunshito':ti,ab OR 'saikokeishito':ti,ab OR 'hochuekkito':ti,ab OR 'shakuyakukanzoto':ti,ab OR 'yokukansan':ti,ab OR 'kamishoyosan':ti,ab) OR (herb*:ti,ab OR plant*:ti,ab OR phytodrug*:ti,ab OR decoction*:ti,ab OR botanical:ti,ab OR phytomedicine:ti,ab) OR (chinese AND adj3 AND (medic*:ti,ab OR herb*:ti,ab OR drug*:ti,ab OR formul*:ti,ab OR plant*:ti,ab OR prescri*:ti,ab)) |
| #9 | ('parkinson disease'/exp OR parkinson*:ab,ti) AND (('herbal medicine'/exp OR 'traditional medicine'/exp OR 'phytotherapy'/exp OR 'medicinal plant'/exp OR 'plant medicinal product'/exp OR 'korean medicine'/exp OR 'herbaceous agent'/exp) OR (tokishakuyakusan:ti,ab OR 'hachimijiogan':ti,ab OR 'goshajinkigan':ti,ab OR 'rikkunshito':ti,ab OR 'saikokeishito':ti,ab OR 'hochuekkito':ti,ab OR 'shakuyakukanzoto':ti,ab OR 'yokukansan':ti,ab OR 'kamishoyosan':ti,ab) OR (herb*:ti,ab OR plant*:ti,ab OR phytodrug*:ti,ab OR decoction*:ti,ab OR botanical:ti,ab OR phytomedicine:ti,ab) OR (chinese AND adj3 AND (medic*:ti,ab OR herb*:ti,ab OR drug*:ti,ab OR formul*:ti,ab OR plant*:ti,ab OR prescri*:ti,ab))) |
| #10 | ('parkinson disease'/exp OR parkinson*:ab,ti) AND (('herbal medicine'/exp OR 'traditional medicine'/exp OR 'phytotherapy'/exp OR 'medicinal plant'/exp OR 'plant medicinal product'/exp OR 'korean medicine'/exp OR 'herbaceous agent'/exp) OR (tokishakuyakusan:ti,ab OR 'hachimijiogan':ti,ab OR 'goshajinkigan':ti,ab OR 'rikkunshito':ti,ab OR 'saikokeishito':ti,ab OR 'hochuekkito':ti,ab OR 'shakuyakukanzoto':ti,ab OR 'yokukansan':ti,ab OR 'kamishoyosan':ti,ab) OR (herb*:ti,ab OR plant*:ti,ab OR phytodrug*:ti,ab OR decoction*:ti,ab OR botanical:ti,ab OR phytomedicine:ti,ab) OR (chinese AND adj3 AND (medic*:ti,ab OR herb*:ti,ab OR drug*:ti,ab OR formul*:ti,ab OR plant*:ti,ab OR prescri*:ti,ab))) AND [humans]/lim |
| #11 | ('parkinson disease'/exp OR parkinson*:ab,ti) AND (('herbal medicine'/exp OR 'traditional medicine'/exp OR 'phytotherapy'/exp OR 'medicinal plant'/exp OR 'plant medicinal product'/exp OR 'korean medicine'/exp OR 'herbaceous agent'/exp) OR (tokishakuyakusan:ti,ab OR 'hachimijiogan':ti,ab OR 'goshajinkigan':ti,ab OR 'rikkunshito':ti,ab OR 'saikokeishito':ti,ab OR 'hochuekkito':ti,ab OR 'shakuyakukanzoto':ti,ab OR 'yokukansan':ti,ab OR 'kamishoyosan':ti,ab) OR (herb*:ti,ab OR plant*:ti,ab OR phytodrug*:ti,ab OR decoction*:ti,ab OR botanical:ti,ab OR phytomedicine:ti,ab) OR (chinese AND adj3 AND (medic*:ti,ab OR herb*:ti,ab OR drug*:ti,ab OR formul*:ti,ab OR plant*:ti,ab OR prescri*:ti,ab))) AND [humans]/lim AND ([controlled clinical trial]/lim OR [randomized controlled trial]/lim) |

(3) [Pubmed]

| #1 | Search (((((("Phytotherapy"[Mesh]) OR ( "Medicine, Traditional"[Mesh] OR "Medicine, Chinese Traditional"[Mesh] OR "Medicine, Korean Traditional"[Mesh] OR "Medicine, East Asian Traditional"[Mesh] )) OR "Herbal Medicine"[Mesh]) OR "Plant Preparations"[Mesh]) OR "Drugs, Chinese Herbal"[Mesh]) OR "Plant Extracts"[Mesh]) OR "Plants, Medicinal"[Mesh] |
| --- | --- |
| #2 | Search (herb* OR plant* OR phytodrug*)[tiab] |
| #3 | Search herb*[tiab] OR plant*[tiab] OR phytodrug*[tiab] |
| #4 | Search Chinese adj3(medic* or herb* or drug* or formul* or plant* or prescri*)[tiab] |
| #5 | Search (Chinese or oriental) adj3 medicine$)[tiab] |
| #6 | Search Chinese adj3 (medic* or herb* or drug* or formul* or plant* or prescri*) Schema: all |
| #7 | Search Chinese adj3 medic$ Schema: all |
| #8 | Search kamishoyosan |
| #9 | Search yokukansan or yoku-kan-san or yi-gan-san |
| #10 | Search yokukansan or yoku-kan-san or yi-gan-san [tiab] |
| #11 | Search shakuyakukanzoto [tiab] |
| #12 | Search hochuekkito [tiab] |
| #13 | Search saikokeishito[tiab] |
| #14 | Search rikkunshito[tiab] |
| #15 | Search goshajinkigan oxaliplatin |
| #16 | Search goshajinkigan[tiab] |
| #17 | Search hachimijiogan [tiab] |
| #18 | Search tokishakuyakusan[tiab] |
| #19 | Search "Parkinson Disease"[Mesh] OR "Parkinsonian Disorders"[Mesh] |
| #20 | Search "Parkinson Disease"[tiab] OR "Parkinsonian Disorders"[tiab] |
| #21 | Search Parkinson*[tiab] |
| #22 | Search ((Parkinson*[tiab]) OR ("Parkinson Disease"[tiab] OR "Parkinsonian Disorders"[tiab])) OR ("Parkinson Disease"[Mesh] OR "Parkinsonian Disorders"[Mesh]) |
| #23 | Search ((((((((((((tokishakuyakusan[tiab]) OR hachimijiogan [tiab]) OR goshajinkigan[tiab]) OR goshajinkigan oxaliplatin) OR rikkunshito[tiab]) OR saikokeishito[tiab]) OR hochuekkito [tiab]) OR shakuyakukanzoto [tiab]) OR (yokukansan or yoku-kan-san or yi-gan-san [tiab])) OR kamishoyosan) OR (Chinese adj3(medic* or herb* or drug* or formul* or plant* or prescri*)[tiab])) OR (herb*[tiab] OR plant*[tiab] OR phytodrug*[tiab])) OR ((((((("Phytotherapy"[Mesh]) OR ( "Medicine, Traditional"[Mesh] OR "Medicine, Chinese Traditional"[Mesh] OR "Medicine, Korean Traditional"[Mesh] OR "Medicine, East Asian Traditional"[Mesh] )) OR "Herbal Medicine"[Mesh]) OR "Plant Preparations"[Mesh]) OR "Drugs, Chinese Herbal"[Mesh]) OR "Plant Extracts"[Mesh]) OR "Plants, Medicinal"[Mesh]) |
| #24 | Search ((((((((((((((tokishakuyakusan[tiab]) OR hachimijiogan [tiab]) OR goshajinkigan[tiab]) OR goshajinkigan oxaliplatin) OR rikkunshito[tiab]) OR saikokeishito[tiab]) OR hochuekkito [tiab]) OR shakuyakukanzoto [tiab]) OR (yokukansan or yoku-kan-san or yi-gan-san [tiab])) OR kamishoyosan) OR (Chinese adj3(medic* or herb* or drug* or formul* or plant* or prescri*)[tiab])) OR (herb*[tiab] OR plant*[tiab] OR phytodrug*[tiab])) OR ((((((("Phytotherapy"[Mesh]) OR ( "Medicine, Traditional"[Mesh] OR "Medicine, Chinese Traditional"[Mesh] OR "Medicine, Korean Traditional"[Mesh] OR "Medicine, East Asian Traditional"[Mesh] )) OR "Herbal Medicine"[Mesh]) OR "Plant Preparations"[Mesh]) OR "Drugs, Chinese Herbal"[Mesh]) OR "Plant Extracts"[Mesh]) OR "Plants, Medicinal"[Mesh]))) AND (((Parkinson*[tiab]) OR ("Parkinson Disease"[tiab] OR "Parkinsonian Disorders"[tiab])) OR ("Parkinson Disease"[Mesh] OR "Parkinsonian Disorders"[Mesh])) Filters: Clinical Trial |
| #25 | Search ((((((((((((((tokishakuyakusan[tiab]) OR hachimijiogan [tiab]) OR goshajinkigan[tiab]) OR goshajinkigan oxaliplatin) OR rikkunshito[tiab]) OR saikokeishito[tiab]) OR hochuekkito [tiab]) OR shakuyakukanzoto [tiab]) OR (yokukansan or yoku-kan-san or yi-gan-san [tiab])) OR kamishoyosan) OR (Chinese adj3(medic* or herb* or drug* or formul* or plant* or prescri*)[tiab])) OR (herb*[tiab] OR plant*[tiab] OR phytodrug*[tiab])) OR ((((((("Phytotherapy"[Mesh]) OR ( "Medicine, Traditional"[Mesh] OR "Medicine, Chinese Traditional"[Mesh] OR "Medicine, Korean Traditional"[Mesh] OR "Medicine, East Asian Traditional"[Mesh] )) OR "Herbal Medicine"[Mesh]) OR "Plant Preparations"[Mesh]) OR "Drugs, Chinese Herbal"[Mesh]) OR "Plant Extracts"[Mesh]) OR "Plants, Medicinal"[Mesh]))) AND (((Parkinson*[tiab]) OR ("Parkinson Disease"[tiab] OR "Parkinsonian Disorders"[tiab])) OR ("Parkinson Disease"[Mesh] OR "Parkinsonian Disorders"[Mesh])) |

(4) [CNKI]

| #1 | 帕金森病 |
| --- | --- |
| #2 | 帕金森氏病 |
| #3 | 震颤麻痹 |
| #4 | 颤病 |
| #5 | 颤证 |
| #6 | 颤震 |
| #7 | 颤拘病 |
| #8 | 振掉 |
| #9 | 拘病 |
| #10 | Parkinson Disease |
| #11 | OR / #1-#10 |
| #12 | 中药 |
| #13 | 中医 |
| #14 | 汤 |
| #15 | 饮 |
| #16 | 散 |
| #17 | 丸 |
| #18 | 中成药 |
| #19 | 方剂 |
| #20 | 中西医结合 |
| #21 | 颗粒 |
| #22 | 胶囊 |
| #23 | 口服液 |
| #24 | OR/ #12-#23 |
| #25 | 补肾养肝OR补肾活血OR补肾平颤OR补督舒经熄风 |
| #26 | 熄风定颤OR熄风止癫OR熄风止痉OR滋阴熄风OR滋补肝肾OR活血熄风OR止痫 |
| #27 | 羚羊角丸OR柴胡疏肝散OR大定风珠 OR 桂枝加葛根湯OR六味地黄丸OR健脾益肾方OR去癫汤OR定振汤 OR定癲饮OR复方抗癲丸OR龟羚帕安丸OR龟羚帕安胶囊 |
| #28 | 帕病1号OR帕病2号OR帕病3号 |
| #29 | 抗震止痉胶囊OR脑康宁 OR通心络胶囊OR蝎蜈胶囊 |
| #30 | 培补肝肾方OR 清心化痰汤 OR柔肝通络汤 OR [熟地平颤汤](http://www.baidu.com/link%5C?url=F-QxZ_nvmveaIFH2ddy9cnweKfSCHdwRlFWABXcdP2b67oxqUVPVUFaPmb2jnRJFxuYyYnREtjblM6uvYOJR1_) OR 坎离汤OR疏筋解毒汤OR五虎追风散OR一贯煎 OR 大补阴丸 OR益元饮 |
| #31 | 随机 |
| #32 | 对照 |
| #33 | OR/ #31-#32 |
| #34 | #11 AND #24 |
| #35 | #34 AND #33 |
| #36 | #11 AND (#24 OR #25) |
| #37 | #36 AND #33 |
| #38 | #11 AND #27 |
| #39 | #38 AND #33 |
| #40 | #11 AND (#28 OR #29) |
| #41 | #36 AND #33 |
| #42 | #11 AND #30 |
| #43 | #42 AND #33 |
| #44 | #35 AND #37 AND #39 AND #41 AND #43 |

(5) [Oriental Medicine Advanced Searching Integrated System (OASIS), National Digital Science Library (NDSL)]

(파킨슨 or parkinson) and 한약

2. Searching strategy for acupuncture

(1) [CENTRAL]

#1 MeSH descriptor: [Parkinson Disease] explode all trees

#2 Parkinson*:ti,ab,kw (Word variations have been searched)

#3 #1 or #2

#4 MeSH descriptor: [Acupuncture] explode all trees

#5 MeSH descriptor: [Acupuncture Analgesia] explode all trees

#6 MeSH descriptor: [Acupuncture Points] explode all trees

#7 MeSH descriptor: [Acupuncture Therapy] explode all trees

#8 MeSH descriptor: [Electroacupuncture] explode all trees

#9 MeSH descriptor: [Acupuncture, Ear] explode all trees

#10 acupuncture or akupuncture or acupoint* or eletroacupuncture or needling or needle* or trigger points:ti,ab,kw (Word variations have been searched)

#11 ((meridian or non-meridian or trigger) near 10 point$):ti,ab,kw (Word variations have been searched)

#12 ((meridian or non-meridian or trigger) near 10 point*):ti,ab,kw (Word variations have been searched)

#13 acupotomy or acup*:ti,ab,kw

#14 #4 or #5 or #6 or #7 or #8 or #9 or #10 or #11 or #12 or #13

#15 #3 and #14

(2) [EMBASE]

#1 'parkinson disease'/exp

#2 parkinson*:ab,ti

#3 'parkinson disease'/exp OR parkinson*:ab,ti

#4 'acupuncture'/exp OR 'acupuncture analgesia'/exp OR 'electroacupuncture'/exp

#5 'acupuncture*':ab,ti OR 'acupuncture analgesia':ab,ti OR 'electroacupuncture':ab,ti OR 'acupuncture points':ab,ti

#6 'acupuncture ear':ab,ti AND needling*:ab,ti OR needle*:ab,ti OR 'tigger point':ab,ti OR akupuncture*

#7 '((meridian or non-meridian or trigger) near 10 point$)':ab,ti

acupotomy OR acup*:ab,ti

#8 ('acupuncture'/exp OR 'acupuncture analgesia'/exp OR 'electroacupuncture'/exp) OR ('acupuncture*':ab,ti OR 'acupuncture analgesia':ab,ti OR 'electroacupuncture':ab,ti OR 'acupuncture points':ab,ti) OR ('acupuncture ear':ab,ti AND needling*:ab,ti OR needle*:ab,ti OR 'tigger point':ab,ti OR akupuncture*) OR '((meridian or non-meridian or trigger) near 10 point$)':ab,ti OR (acupotomy OR acup*:ab,ti)

#9 ('parkinson disease'/exp OR parkinson*:ab,ti) AND (('acupuncture'/exp OR 'acupuncture analgesia'/exp OR 'electroacupuncture'/exp) OR ('acupuncture*':ab,ti OR 'acupuncture analgesia':ab,ti OR 'electroacupuncture':ab,ti OR 'acupuncture points':ab,ti) OR ('acupuncture ear':ab,ti AND needling*:ab,ti OR needle*:ab,ti OR 'tigger point':ab,ti OR akupuncture*) OR '((meridian or non-meridian or trigger) near 10 point$)':ab,ti OR (acupotomy OR acup*:ab,ti)) AND [humans]/lim

#10 ('parkinson disease'/exp OR parkinson*:ab,ti) AND (('acupuncture'/exp OR 'acupuncture analgesia'/exp OR 'electroacupuncture'/exp) OR ('acupuncture*':ab,ti OR 'acupuncture analgesia':ab,ti OR 'electroacupuncture':ab,ti OR 'acupuncture points':ab,ti) OR ('acupuncture ear':ab,ti AND needling*:ab,ti OR needle*:ab,ti OR 'tigger point':ab,ti OR akupuncture*) OR '((meridian or non-meridian or trigger) near 10 point$)':ab,ti OR (acupotomy OR acup*:ab,ti))

(3) [Pubmed]

#1 Search "Parkinson Disease"[tiab] OR "Parkinson*"[tiab]

#2 Search "Parkinson Disease"[Mesh]

#3 Search ("Parkinson Disease"[Mesh]) OR ("Parkinson Disease"[tiab] OR "Parkinson*"[tiab])

#4 Search "Acupuncture"[Mesh] OR "Acupuncture Therapy"[Mesh] OR "Acupuncture, Ear"[Mesh] OR "Acupuncture Points"[Mesh] OR "Acupuncture Analgesia"[Mesh]

#5 Search "Acupuncture"[tiab] OR "Acupuncture Therapy"[tiab] OR "Acupuncture, Ear"[tiab] OR "Acupuncture Points"[tiab] OR "Acupuncture Analgesia"[tiab]

#6 Search akupuncture[tiab] or acupoint*[tiab] or eletroacupuncture[tiab] or needling[tiab] or needle*[tiab] or "trigger points"[tiab] or acupotomy[tiab] or acup*[tiab]

#7 Search meridian or non-meridian or trigger) near10 point$

#8 Search (meridian or non-meridian or trigger) near10 point*

#9 Search ((((((meridian or non-meridian or trigger) near10 point*)) OR (meridian or non-meridian or trigger) near10 point$)) OR (akupuncture[tiab] or acupoint*[tiab] or eletroacupuncture[tiab] or needling[tiab] or needle*[tiab] or "trigger points"[tiab] or acupotomy[tiab] or acup*[tiab])) OR ("Acupuncture"[tiab] OR "Acupuncture Therapy"[tiab] OR "Acupuncture, Ear"[tiab] OR "Acupuncture Points"[tiab] OR "Acupuncture Analgesia"[tiab])) OR ("Acupuncture"[Mesh] OR "Acupuncture Therapy"[Mesh] OR "Acupuncture, Ear"[Mesh] OR "Acupuncture Points"[Mesh] OR "Acupuncture Analgesia"[Mesh])

#10 Search ((((((((meridian or non-meridian or trigger) near10 point*)) OR (meridian or non-meridian or trigger) near10 point$)) OR (akupuncture[tiab] or acupoint*[tiab] or eletroacupuncture[tiab] or needling[tiab] or needle*[tiab] or "trigger points"[tiab] or acupotomy[tiab] or acup*[tiab])) OR ("Acupuncture"[tiab] OR "Acupuncture Therapy"[tiab] OR "Acupuncture, Ear"[tiab] OR "Acupuncture Points"[tiab] OR "Acupuncture Analgesia"[tiab])) OR ("Acupuncture"[Mesh] OR "Acupuncture Therapy"[Mesh] OR "Acupuncture, Ear"[Mesh] OR "Acupuncture Points"[Mesh] OR "Acupuncture Analgesia"[Mesh]))) AND (("Parkinson Disease"[Mesh]) OR ("Parkinson Disease"[tiab] OR "Parkinson*"[tiab]))

(4) [CNKI]

#1 帕金森病

#2 帕金森氏病

#3 震颤麻痹

#4 颤病

#5 颤证

#6 颤震

#7 颤拘病

#8 振掉

#9 拘病 #10 Parkinson Disease

#11 OR / #1-#10

#12 针刺

#13 针灸

#14 电针

#15 刺法

#16 针

#17 耳针

#18 穴位注射

#19 药针

#20 蜂针

#21 Point injection

#22 温针

#23 刀针

#24 OR/ #12-#23

#25 随机

#26 对照

#27 系统评价

#28 Meta分析

#29 OR/#24-#28

#30 #11 AND #23 AND #29

(5) [Oriental Medicine Advanced Searching Integrated System (OASIS), National Digital Science Library (NDSL)]

파킨슨 and 침

3. Searching strategy for moxibustion

(1) [CENTRAL]

| #1 | MeSH descriptor: [Parkinson Disease] explode all trees |
| --- | --- |
| #2 | Parkinson*:ti,ab,kw (Word variations have been searched) |
| #3 | #1 or #2 |
| #4 | moxibustion":ti,ab,kw (Word variations have been searched) |
| #5 | mox*:ti,ab,kw (Word variations have been searched) |
| #6 | moxibustion*:ti,ab,kw (Word variations have been searched) |
| #7 | meridian:ti,ab,kw (Word variations have been searched) |
| #8 | #4 or #5 or #6 or #7 |
| #9 | #3 and #8 |

(2) [EMBASE]

| #1 | 'parkinson disease'/exp |
| --- | --- |
| #2 | parkinson*:ab,ti |
| #3 | 'parkinson disease'/exp OR parkinson*:ab,ti |
| #4 | 'moxibustion':ab,ti OR mox*:ab,ti OR moxibustion*:ab,ti OR meridian:ab,ti |
| #5 | #3 AND #4 |

(3) [Pubmed]

| #1 | Search "Parkinson Disease"[tiab] OR "Parkinson*"[tiab] |
| --- | --- |
| #2 | Search "Parkinson Disease"[Mesh] |
| #3 | Search ("Parkinson Disease"[Mesh]) OR ("Parkinson Disease"[tiab] OR "Parkinson*"[tiab]) |
| #4 | Search mox*[tiab] or moxibustion*[tiab] or meridian*[tiab] |
| #5 | Search ((mox*[tiab] or moxibustion*[tiab] or meridian*[tiab])) AND (("Parkinson Disease"[Mesh]) OR ("Parkinson Disease"[tiab] OR "Parkinson*"[tiab])) |

(4) [CNKI]

| #1 | 帕金森病 |
| --- | --- |
| #2 | 帕金森氏病 |
| #3 | 震颤麻痹 |
| #4 | 颤病 |
| #5 | 颤证 |
| #6 | 颤震 |
| #7 | 颤拘病 |
| #8 | 振掉 |
| #9 | 拘病 |
| #10 | Parkinson Disease |
| #11 | OR / #1-#10 |
| #12 | 艾灸 |
| #13 | 直接灸 |
| #14 | 间接灸 |
| #15 | 隔物灸 |
| #16 | 隔药灸 |
| #17 | 隔药饼灸 |
| #18 | 督灸 |
| #19 | 温和灸 |
| #20 | 热敏灸 |
| #21 | 药线点灸 |
| #22 | 激光灸 |
| #23 | Moxibustion |
| #24 | Laser moxibustion |
| #25 | OR/ #12-#24 |
| #26 | #6 AND #25 |

(5) [Oriental Medicine Advanced Searching Integrated System (OASIS), National Digital Science Library (NDSL)]

파킨슨 and 뜸

4. Searching strategy for pharmacoacupuncture

(1) [CENTRAL]

| #1 | MeSH descriptor: [Parkinson Disease] explode all trees |
| --- | --- |
| #2 | Parkinson*:ti,ab,kw (Word variations have been searched) |
| #3 | #1 or #2 |
| #4 | MeSH descriptor: [Bee Venoms] explode all trees |
| #5 | bee venom* acupuncture:ti,ab,kw (Word variations have been searched) |
| #6 | bee venom* therapy:ti,ab,kw (Word variations have been searched) |
| #7 | "bee sting* therapy":ti,ab,kw (Word variations have been searched) |
| #8 | "bee venom" or "bee venom*" or apitoxin or apitherapy:ti,ab,kw (Word variations have been searched) |
| #9 | ("acupuncture point injection" or "acupoint injcetion" or acup*) and herb*:ti,ab,kw (Word variations have been searched) |
| #10 | "bee sting" or "bee sting*":ti,ab,kw (Word variations have been searched) |
| #11 | "herbal injection":ti,ab,kw (Word variations have been searched) |
| #12 | #4 or #5 or #6 or #7 or #8 or #9 or #10 or #11 |
| #13 | #3 and #12 |

(2) [EMBASE]

| #1 | 'parkinson disease'/exp |
| --- | --- |
| #2 | parkinson*:ab,ti |
| #3 | 'parkinson disease'/exp OR parkinson*:ab,ti |
| #4 | 'bee venom'/exp |
| #5 | 'bee venom*':ab,ti OR 'pharmacopuncture*':ab,ti OR 'bee venom* acupuncture':ab,ti OR 'bee venom* therapy':ab,ti OR 'bee sting* therapy':ab,ti OR 'bee sting*':ab,ti OR apitoxin OR apitherapy |
| #6 | 'acupuncture point injection':ab,ti OR 'acupoint injcetion':ab,ti OR acup*:ab,ti AND herb*:ab,ti |
| #7 | 'herbal injection*':ab,ti |
| #8 | 'bee venom'/exp OR ('bee venom*':ab,ti OR 'pharmacopuncture*':ab,ti OR 'bee venom* acupuncture':ab,ti OR 'bee venom* therapy':ab,ti OR 'bee sting* therapy':ab,ti OR 'bee sting*':ab,ti OR apitoxin OR apitherapy) OR ('acupuncture point injection':ab,ti OR 'acupoint injcetion':ab,ti OR acup*:ab,ti AND herb*:ab,ti) OR 'herbal injection*':ab,ti |
| #9 | ('parkinson disease'/exp OR 'parkinson*':ab,ti) AND ('bee venom'/exp OR ('bee venom*':ab,ti OR 'pharmacopuncture*':ab,ti OR 'bee venom* acupuncture':ab,ti OR 'bee venom* therapy':ab,ti OR 'bee sting* therapy':ab,ti OR 'bee sting*':ab,ti OR apitoxin OR apitherapy) OR ('acupuncture point injection':ab,ti OR 'acupoint injcetion':ab,ti OR acup*:ab,ti AND herb*:ab,ti) OR 'herbal injection*':ab,ti) |

(3) [Pubmed]

| #1 | Search "Parkinson Disease"[tiab] OR "Parkinson*"[tiab] |
| --- | --- |
| #2 | Search "Parkinson Disease"[Mesh] |
| #3 | Search ("Parkinson Disease"[Mesh]) OR ("Parkinson Disease"[tiab] OR "Parkinson*"[tiab]) |
| #4 | Search "Bee Venoms"[Mesh] |
| #5 | Search "Bee Venom*"[tiab] or apitoxin[tiab] or apitherapy[tiab] or "bee venom* acupuncture"[tiab] or "bee venom* therapy"[tiab] or "bee sting* therapy"[tiab] or "bee sting*"[tiab] or "herbal injection"[tiab] |
| #6 | Search (((("acupuncture point injection"[tiab] or "acupoint injcetion"[tiab] or acup*[tiab]) and herb*[tiab])) OR ("Bee Venom*"[tiab] or apitoxin[tiab] or apitherapy[tiab] or "bee venom* acupuncture"[tiab] or "bee venom* therapy"[tiab] or "bee sting* therapy"[tiab] or "bee sting*"[tiab] or "herbal injection"[tiab])) OR "Bee Venoms"[Mesh] |
| #7 | Search (((((("acupuncture point injection"[tiab] or "acupoint injcetion"[tiab] or acup*[tiab]) and herb*[tiab])) OR ("Bee Venom*"[tiab] or apitoxin[tiab] or apitherapy[tiab] or "bee venom* acupuncture"[tiab] or "bee venom* therapy"[tiab] or "bee sting* therapy"[tiab] or "bee sting*"[tiab] or "herbal injection"[tiab])) OR "Bee Venoms"[Mesh])) AND (("Parkinson Disease"[Mesh]) OR ("Parkinson Disease"[tiab] OR "Parkinson*"[tiab])) |

(4) [CNKI]

| #1 | 帕金森病 |
| --- | --- |
| #2 | 帕金森氏病 |
| #3 | 震颤麻痹 |
| #4 | 颤病 |
| #5 | 颤证 |
| #6 | 颤震 |
| #7 | 颤拘病 |
| #8 | 振掉 |
| #9 | 拘病 |
| #10 | Parkinson Disease |
| #11 | OR/ #1-#10 |
| #12 | 穴位注射 |
| #13 | 药针 |
| #14 | 蜂针 |
| #15 | Point injection |
| #16 | OR/ #12-#15 |
| #17 | 11 AND #16 |

(5) [Oriental Medicine Advanced Searching Integrated System (OASIS), National Digital Science Library (NDSL)]

파킨슨 and 약침

5. Searching strategy for Qigong and Tai chi

(1) [CENTRAL]

#1 MeSH descriptor: [Parkinson Disease] explode all trees

#2 Parkinson*:ti,ab,kw (Word variations have been searched)

#3 #1 or #2

#4 MeSH descriptor: [Qigong] explode all trees

#5 'qi gong' or 'qigong' or chigung or 'chi chung' or 'chi kung':ti,ab,kw (Word variations have been searched)

#6 MeSH descriptor: [Tai Ji] explode all trees

#7 'Tai chi' or Taijiquan or 'Tai-ji':ti,ab,kw (Word variations have been searched) 718

#8 #24 or #25 or #26 or #27

#9 #3 and #8

(2) [EMBASE]

#1 'parkinson disease'/exp

#2 parkinson*:ab,ti

#3 'parkinson disease'/exp OR parkinson*:ab,ti

#4 'qigong'/exp OR 'tai chi'/exp

#5 'qigong':ti,ab OR 'tai chi':ti,ab OR taijiquan:ab,ti OR 'tai-ji':ab,ti

#6 'qi gong':ab,ti OR 'qigong':ab,ti OR chigung:ab,ti OR 'chi chung':ab,ti OR 'chi kung':ab,ti

#7 #10 OR #11 OR #12

#8 #6 AND #13

(3) [Pubmed]

#1 Search "Parkinson Disease"[tiab] OR "Parkinson*"[tiab]

#2 Search "Parkinson Disease"[Mesh]

#3 Search ("Parkinson Disease"[Mesh]) OR ("Parkinson Disease"[tiab] OR "Parkinson*"[tiab])

#4 Search (Qigong[MeSH Terms]) OR Tai Ji[MeSH Terms]

#5 Search Qigong[tiab] OR "Tai Ji"[tiab] OR "qi gong"[tiab] or "qigong"[tiab] or chigung[tiab] or "chi chung"[tiab] or "chi kung"[tiab]

#6 Search "Tai chi"[tiab] or Taijiquan[tiab] or "Tai-ji"[tiab]

#7 Search ((("Tai chi"[tiab] or Taijiquan[tiab] or "Tai-ji"[tiab])) OR (Qigong[tiab] OR "Tai Ji"[tiab] OR "qi gong"[tiab] or "qigong"[tiab] or chigung[tiab] or "chi chung"[tiab] or "chi kung"[tiab])) OR ((Qigong[MeSH Terms]) OR Tai Ji[MeSH Terms])

#8 Search ((((("Tai chi"[tiab] or Taijiquan[tiab] or "Tai-ji"[tiab])) OR (Qigong[tiab] OR "Tai Ji"[tiab] OR "qi gong"[tiab] or "qigong"[tiab] or chigung[tiab] or "chi chung"[tiab] or "chi kung"[tiab])) OR ((Qigong[MeSH Terms]) OR Tai Ji[MeSH Terms]))) AND (("Parkinson Disease"[Mesh]) OR ("Parkinson Disease"[tiab] OR "Parkinson*"[tiab]))

(4) [CNKI]

#1 帕金森病

#2 帕金森氏病

#3 震颤麻痹

#4 颤病

#5 颤证

#6 颤震

#7 颤拘病

#8 振掉

#9 拘病

#10 Parkinson Disease

#11 OR/ #1-#10

#12 气功

#13 qigong

#14 Qi gong

#15 太极拳

#16 Tai Chi

#17 瑜伽

#18 Yoga

#19 OR/ #12-#18

#20 11 AND #19

(5) [Oriental Medicine Advanced Searching Integrated System (OASIS), National Digital Science Library (NDSL)]

파킨슨병 AND 태극권 OR 기공

Supplementary Table 1: Delphi consensus process

| Date | Step | Remarks |
| --- | --- | --- |
| 2017. 07. 10 – 2017. 07. 18 | Organizing 9 experts for Delphi consensus | Four professors of colleges of Korean Medicine  Two primary care physicians  Two methodological specialists  One experts from the Society of Korean Medicine |
| 2017. 07. 18 – 2017. 07. 19 | Make Delphi survey platform using survey monkey |  |
| 2017. 07. 19 – 2017. 07. 24 | 1st round of Delphi survey | Nine respondents (100%) |
| 2017. 07. 25 – 2017. 08. 05 | Analysis of the result of 1st round of Delphi survey and amendment of recommendations for further Delphi survey |  |
| 2017. 07.07 – 2017. 08. 10 | 2nd round of Delphi survey | Nine respondents (100%) |
| 2017. 0. 11 – 2017. 08. 16 | Analysis of the result of 2nd round of Delphi survey and finalizing recommendations |  |

Supplementary Table 2.1: Summary of findings table: Herbal medicines with anti-parkinsonianism drugs for idiopathic Parkinson’s disease

| Outcome (clinical importance) | Total number of patients (number of included studies) | Level of evidence (GRADE) | Relative risks (95% CI) | Anticipated absolute effects (95% CI) | | Comments |
| --- | --- | --- | --- | --- | --- | --- |
|  |  |  |  | Controlled group | Intervention group |  |
| UPDRS total score (critical) | 5330 (75RCTs) | ⊕⊕⊝⊝  Low^a^ |  |  | MD -6.06, 95% CI [-6.82, -5.3] | Lower score suggesting better effect |
| UPDRSﾠⅠ score (critical) | 3100 (44 RCTs) | ⊕⊕⊝⊝  Low^a^ |  |  | MD -1.6, 95% CI [-1.94, -1.26] | Lower score suggesting better effect |
| UPDRS Ⅱ score (critical) | 4311 (59 RCTs) | ⊕⊕⊝⊝  Low^a^ |  |  | MD -2.22, 95% CI [-2.67, -1.76] | Lower score suggesting better effect |
| UPDRS Ⅲ score (critical) | 4909 (70 RCTs) | ⊕⊕⊝⊝  Low^a^ |  |  | MD -3.41, 95% CI [-4.23, -2.59] | Lower score suggesting better effect |
| UPDRS Ⅳ score (critical) | 3078 (40 RCTs) | ⊕⊕⊝⊝  Low^a^ |  |  | MD -1.41, 95% CI [-1.72, -1.10] | Lower score suggesting better effect |
| PDQ-39 summary index (important) | 1490 (20 RCTs) | ⊕⊕⊝⊝  Low^a^ |  |  | MD -9.29, 95% CI [-10.83, -7.75] | Lower score suggesting better effect |
| Levodopa consumption (critical) | 2043 (27 RCTs) | ⊕⊝⊝⊝  Insufficient^a,b^ |  |  | SMD -0.77, 95% CI [-0.99, -0.56] | Lower score suggesting decreased dopamine usage |
| Total adverse event rate (critical) | 3463 (49 RCTs) | ⊕⊕⊝⊝  Low^a^ | RR 0.44 [0.37, 0.52] |  | 113 fewer per 1000 [90 less to 131 less] | Fewer events suggesting fewer adverse events rate |

CI: confidence interval; GRADE: The Grading of Recommendations Assessment, Development and Evaluation; MD: mean difference; RCT: randomized controlled trial; RR: relative risk; SMD: standard mean difference; UPDRS: Unified Parkinson’s Disease Rating Scale; PDQ-39: Parkinson’s Disease Questionnaire-39; a: Downgraded twice due to unclear risk of bias in the sequence generation and allocation concealment domains in most studies. In addition, there were also concerns that blinding of participants and personnel was not possible in the nature of the intervention (moxibustion) itself; b: Downgraded once due to significant statistical heterogeneity

Supplementary Table 2.2: Summary of findings table: Bosin-yanggan-sigpung-bang with anti-parkinsonianism drugs for idiopathic Parkinson’s disease

| Outcome (clinical importance) | Total number of patients (number of included studies) | Level of evidence (GRADE) | Relative risks (95% CI) | Anticipated absolute effects (95% CI) | | Comments |
| --- | --- | --- | --- | --- | --- | --- |
|  |  |  |  | Controlled group | Intervention group |  |
| UPDRS total score (critical) | 289 (4 RCTs) | ⊕⊝⊝⊝  Insufficient^a,b,c^ |  |  | MD - 11.39, 95% CI [-16.2, -6.57] | Lower score suggesting better effect |
| Levodopa consumption (critical) | 289 (4 RCTs) | ⊕⊝⊝⊝  Insufficient^a,b,c^ |  |  | SMD - 1.04, 95% CI [-1.49, -0.58] | Lower score suggesting decreased dopamine usage |

CI: confidence interval; GRADE: The Grading of Recommendations Assessment, Development and Evaluation; MD: mean difference; SMD: standard mean difference; RCT: randomized controlled trial; UPDRS: Unified Parkinson’s Disease Rating Scale; a: Downgraded twice due to unclear risk of bias in participants and outcome assessor blinding in most studies; b: Downgraded once due to significant statistical heterogeneity; c: Downgraded due to small sample size

References

1. 魏风朱小晓, "补肾养肝熄风方药协同美多巴治疗帕金森病疗效观察," 辽宁中医杂志, no. 11, 2012.

2. H. Wang, M. Yang, Y. Liu, S. Li, andM. Li, "Effectiveness of Bushen Huoxue Granule (补肾活血颗粒) on5-serotonin and norepinephrine in the brain of Parkinson's disease patientswith depressive state," in Chinesejournal of integrative medicine, pp. 944-948: 2014.

3. 王德刚, 陆征宇, and 赵虹, "补肾养肝熄风方药治疗肝肾阴虚型帕金森病临床研究," 辽宁中医杂志, no. 08, 2012.

4. 周洋, 王德刚, 陆征宇, 汪涛, and 赵虹, "补肾养肝熄风法治疗肝肾阴虚型帕金森病30例," 辽宁中医杂志, no. 05, 2013.

Supplementary Table 2.3: Summary of findings table: Bosin-hwalhyeol-cheobang with anti-parkinsonianism drugs for idiopathic Parkinson’s disease

| Outcome (clinical importance) | Total number of patients (number of included studies) | Level of evidence (GRADE) | Relative risks (95% CI) | Anticipated absolute effects (95% CI) | | Comments |
| --- | --- | --- | --- | --- | --- | --- |
|  |  |  |  | Controlled group | Intervention group |  |
| UPDRS total score (critical) | 353 (6 RCTs) | ⊕⊕⊕⊝  Moderate^a^ |  |  | MD -6.32, 95% CI [-8.6, -4.05] | Lower score suggesting better effect |
| PDQ-39 (important) | 177 (2 RCTs) | ⊕⊕⊝⊝  Low^a,c^ |  |  | MD -9.01, 95% CI [-11.91, 6.11] | Lower score suggesting better effect |
| Total adverse event rate (critical) | 290 (3 RCTs) | ⊕⊕⊕⊝  Moderate^c^ | RR 0.46, 95% CI [0.21, 1.03] |  | 24 fewer per 1000 [32 less, 41 more] | Fewer events suggesting fewer adverse events rate |

CI: confidence interval; GRADE: The Grading of Recommendations Assessment, Development and Evaluation; MD: mean difference; RCT: randomized controlled trial; RR: relative risk; UPDRS: Unified Parkinson’s Disease Rating Scale; a: Downgraded twice due to unclear risk of bias in participants and outcome assessor blinding in most studies; b: Downgraded once due to significant statistical heterogeneity; c: Downgraded due to small sample size

References

1. 安畅, "自拟补肾活血汤治疗帕金森病30例临床观察," 中医药导报, no. 06, 2013.

2. 窦维华, 刁丽梅, "补肾活血汤治疗帕金森病的临床研究," 长春中医药大学学报, no. 04, 2010.

3. 郭云霞, 李绍旦, and 杨明会, "补肾活血颗粒治疗帕金森病抑郁临床研究," 环球中医药, no. 04, 2014.

4. 李敏, "补肾活血法治疗帕金森病的临床研究,"2010.

5. 李军艳, 石桦, 陈美南, 唐明, and 杨秀丽, "补肾活血法对强直少动型帕金森病患者生活质量的影响," 实用医药杂志, no.05, 2012.

6. 李敏 and 杨明会, "补肾活血法治疗帕金森病患者30例临床研究," 西部中医药, no. 03, 2013.

7. 牛小英, "补肾活血汤治疗帕金森病的临床研究," 中国当代医药, no. 14, 2014.

8. 肖晓岚, "补肾活血颗粒治疗帕金森病临床研究,"2010.

9. 杨明会, 李敏, 窦永起, et al.,"补肾活血颗粒对帕金森病患者运动功能的影响:多中心、随机、双盲、安慰剂对照研究," 中西医结合学报, no. 03, 2010.

10. 张鑫, "补肾活血方治疗帕金森病的临床对照研究,"2013. Supplementary Table 2.4: Summary of findings table: Bosin-hwalhyeol-tonglag-cheobang with anti-parkinsonianism drugs for idiopathic Parkinson’s disease

| Outcome (clinical importance) | Total number of patients (number of included studies) | Level of evidence (GRADE) | Relative risks (95% CI) | Anticipated absolute effects (95% CI) | | Comments |
| --- | --- | --- | --- | --- | --- | --- |
|  |  |  |  | Controlled group | Intervention group |  |
| UPDRS total score (critical) | 76 (1 RCT) | ⊕⊕⊝⊝  Low^a,b^ |  |  | MD -15.40, 95% CI [-19.80, -11.00] | Lower score suggesting better effect |
| PDQ-39 (important) | 218 (2 RCTs) | ⊕⊕⊝⊝  Low^a,b^ |  |  | MD -7.05, 95% CI [-11.80, -2.30] | Lower score suggesting better effect |

CI: confidence interval; GRADE: The Grading of Recommendations Assessment, Development and Evaluation; MD: mean difference; RCT: randomized controlled trial; UPDRS: Unified Parkinson’s Disease Rating Scale; a: Downgraded once due to unclear risk of bias in participants and outcome assessor blinding in most studies; b: Downgraded due to small sample size

References

1. 潘立强, "补肾活血通络中药辅助治疗原发性帕金森病的疗效评价," 航空航天医学杂志, no. 09, 2016.

2. 王彦, "补肾活血通络胶囊治疗原发性帕金森病临床疗效观察," 中国继续医学教育, no.17, 2015.

3. 仲诚, 黄萍, 孙照国, and 王程, "补肾活血通络胶囊治疗原发性帕金森病120例," 中国实验方剂学杂志, no. 24, 2012.

Supplementary Table 2.5: Summary of findings table: Sugji-pyeongjeon-tang with anti-parkinsonianism drugs for idiopathic Parkinson’s disease

| Outcome (clinical importance) | Total number of patients (number of included studies) | Level of evidence (GRADE) | Relative risks (95% CI) | Anticipated absolute effects (95% CI) | | Comments |
| --- | --- | --- | --- | --- | --- | --- |
|  |  |  |  | Controlled group | Intervention group |  |
| UPDRS Ⅱ score (critical) | 220 (3 RCTs) | ⊕⊕⊝⊝  Low^a,b^ |  |  | MD -1.59, 95% CI [-2.51, -0.67] | Lower score suggesting better effect |
| UPDRS Ⅲ score (critical) | 220 (3 RCTs) | ⊕⊕⊝⊝  Low^a,b^ |  |  | MD -2.51, 95% CI [-3.89, -1.13] | Lower score suggesting better effect |
| Levodopa consumption (important) | 160 (2 RCTs) | ⊕⊕⊝⊝  Low^a,b^ |  |  | MD -0.05, 95% CI [-0.26, 0.36] | Lower score suggesting better effect |
| Total adverse event rate (critical) | 160 (3 RCTs) | ⊕⊕⊝⊝  Low^a,b^ | RR 0.80, 95% CI [0.25, 2.52] |  | 50 less per 1000, [188 less, 380 more] | Fewer events suggesting fewer adverse events rate |

CI: confidence interval; GRADE: The Grading of Recommendations Assessment, Development and Evaluation; MD: mean difference; RCT: randomized controlled trial; RR: relative risk; UPDRS: Unified Parkinson’s Disease Rating Scale; a: Downgraded once due to unclear risk of bias in participants and outcome assessor blinding in most studies; b: Downgraded due to small sample size

Reference

1. 陈顺中, 袁灿兴, 常华军, 吴年宝, 全亚萍, and 袁成业, "滋补肝肾通络解毒法对美多巴治疗帕金森病增效减毒作用的临床研究," 江苏中医药, no. 12, 2012.

2. 袁灿兴, 支惠萍, 陈顺中, et al.,"熟地平颤汤结合西医常规疗法治疗帕金森病的临床多中心随机对照研究," 上海中医药杂志, no. 06, 2010.

3. 周洁, 叶青, and 袁灿兴, "熟地平颤汤治疗帕金森氏病的随机对照研究," 中华中医药学刊, no.06, 2014.

Supplementary Table 3.1: Summary of findings table: combination treatment with anti-parkinsonianism drugs and acupuncture for idiopathic Parkinson’s disease

| Outcome (clinical importance) | Total number of patients (number of included studies) | Level of evidence (GRADE) | Relative risks (95% CI) | Anticipated absolute effects (95% CI) | | Comments |
| --- | --- | --- | --- | --- | --- | --- |
|  |  |  |  | Controlled group | Intervention group |  |
| Overall clinical effectiveness (important) | 1371 (21 RCTs) | ⊕⊕⊝⊝ Low | RR 1.2, 95% CI [1.08, 1.33] |  | 650 more per 1000 | Higher score suggesting better effect |
| Webster scale (Critical) | 354 (7 RCTs) | ⊕⊕⊝⊝ Low |  |  | MD -3.09, 95% CI [-4.8, -1.38] | Lower score suggesting better effect |
| UPDRS total score (critical) | 890 (15 RCT) | ⊕⊕⊝⊝ Low |  |  | MD -6.72, 95% CI [-10.24, -3.2] | Lower score suggesting better effect |

CI: confidence interval; GRADE: The Grading of Recommendations Assessment, Development and Evaluation; MD: mean difference; RCT: randomized controlled trial; UPDRS: Unified Parkinson’s Disease Rating Scale

Reference

1. 陈秀华, 李漾奎瑜, 腹针配合美多巴治疗帕金森氏病临床观察. 中国针灸2007;27:562-564.

2. 任晓明, 石炎, 宋双临, 胡晓晴韩宗华, 补益肝肾法针刺治疗帕金森病的临床观察. 中华中医药学刊2011;29:2470-2473.

3. 韩天文, 刘建明, 李艳景, 醒脑开窍针刺法改善震颤麻痹的临床观察.川北医学院学2011;26:136-138.

4. 刘肖瑜, 姜拯坤, 针药并用治疗帕金森病临床观察. 上海针灸杂志2013;32:461-463.

5. 李云龙, 针刺联合美多芭治疗帕金森病的120例临床研究. 中外医疗2015:120-121.

6. 林燕, 刘梅, 针刺联合药物治疗帕金森病31例. 河南中医2015;35:1430-1432.

7. 张少雷, 药物配合针刺治疗帕金森病疗效观察. 实用中医药杂志2016;32:230-231.

8. Fu B, Lun X, Rong LLin WD, Electroacupuncture at head and du pulse acpoints for treatment of Parkinson disease: Randomized controlled observation. Chinese Journal of Clinical Rehabilitation2004;8:4524-4525.

9. 杨秀毅, 电针刺激联合左旋多巴治疗帕金森病的疗效观察. 中国实用神经疾病杂志2016;19:103-105.

10. 王晓平, 罗永杰, 吴景芬, 李晓佳陈凯, 多巴丝肼联合电针对老年帕金森病患者血清白细胞介素-1β、胱抑素c及血清离子水平的影响. 中国老年学杂志2016;36:4301-4303.

11. Zhang WG, Wang GBQin Y, Scalp acupuncture for treatment of parkinson disease: 32case. Guangming J Chin Med2002;17:55-57.

12. 杨焱陈, 头针治疗帕金森病30例的临床观察. 针灸临床杂志2004;20:36.

13. 刘丹, 刘芳邵滢如, 针刺舞蹈震颤控制区配合药物治疗帕金森病疗效观察. 上海针灸杂志2015;34:825-826.

14. 常学辉, 张良芝李彦杰, 针药结合治疗帕金森病疗效观察. 中国针灸2008;28:645-647.

15. 顾侃, 刘昆, 陆征宇, 樊晓鹏宗蕾, 针药并用治疗帕金森病临床观察. 上海针灸杂志2013;32:993-995.

16. 李海悦王颖, 通督调神针刺法治疗血管性帕金森综合征的临床研究. 云南中医学院学报2016;39:44-46.

17. 孙赫楠, 针药结合治疗帕金森病32例疗效观察. 内蒙古中医药2014:10-11.

18. 索庆芳, 王丽晔彭明华, 电项针结合电头针治疗帕金森病70例疗效观察. 中国中医基础医学杂志2015;21:860-861,883.

19. Yang DH, Shi YJia YM, Influence of acupuncture plus drug in the amelioration of symptoms and blood antioxidant system of patients with Parkinson disease. Chinese Journal of Clinical Rehabilitation2006;10:14-16.

20. 杨丹红, 陈华德方针, 针刺配合药物对帕金森病康复治疗的疗效观察. 针灸临床杂志2006;22:16-18.

21. Zhuang XWang L, Acupuncture Treatment of Parkinson’s Disease—A Report of 29 Cases. Periodical 2000;20:265-267. Supplementary Table 3.2: Summary of findings table: combination treatment with anti-parkinsonianism drugs and manual acupuncture for idiopathic Parkinson’s disease

| Outcome  (Clinical importance) | Total number of patients (number of included studies) | Level of evidence (GRADE) | Relative risks (95% CI) | Anticipated absolute effects (95% CI) | | Comments |
| --- | --- | --- | --- | --- | --- | --- |
|  |  |  |  | Controlled group | Intervention group |  |
| Webster scale (critical) | 62 (1 RCT) | ⊕⊕⊕⊝ Moderate |  |  | MD -3.75, 95% CI [-5.27, -2.23] | Lower score suggesting better effect |

CI: confidence interval; GRADE: The Grading of Recommendations Assessment, Development and Evaluation; MD: mean difference; RCT: randomized controlled trial

References

1. 陈秀华, 李漾奎瑜, 腹针配合美多巴治疗帕金森氏病临床观察.中国针灸2007;27:562-564.

2. 任晓明, 石炎, 宋双临, 胡晓晴韩宗华, 补益肝肾法针刺治疗帕金森病的临床观察.中华中医药学刊 2011;29:2470-2473.

3. 韩天文, 刘建明李艳景, 醒脑开窍针刺法改善震颤麻痹的临床观察.川北医学院学报2011;26:136-138.

4. 刘肖瑜姜拯坤, 针药并用治疗帕金森病临床观察.上海针灸杂志2013;32:461-463.

5. 李云龙, 针刺联合美多芭治疗帕金森病的120例临床研究.中外医疗2015:120-121.

6. 林燕, 刘梅, 针刺联合药物治疗帕金森病31例.河南中医2015;35:1430-1432.

7. 张少雷, 药物配合针刺治疗帕金森病疗效观察.实用中医药杂志2016;32:230-231.

Supplementary Table 3.3: Summary of findings table: combination treatment with anti-parkinsonianism drugs and electroacupuncture for idiopathic Parkinson’s disease

| Outcome (Clinical importance) | Total number of patients (number of included studies) | Level of evidence (GRADE) | Relative risks (95% CI) | Anticipated absolute effects (95% CI) | | Comments |
| --- | --- | --- | --- | --- | --- | --- |
|  |  |  |  | Controlled group | Intervention group |  |
| Overall clinical effectiveness (important) | 104 (2 RCTs) | ⊕⊕⊕⊝ Moderate | RR 1.22, 95% CI [0.91, 1.63] |  | 155 more per 1000 | Higher score suggesting better effect |
| Webster scale (critical) | 104 (2 RCTs) | ⊕⊕⊝⊝ Low |  |  | MD -2.98, 95% CI [-6.11, 0.15] | Lower score suggesting better effect |
| UPDRS total score (critical) | 101 (2 RCTs) | ⊕⊕⊕⊝ Moderate |  |  | MD 3.8, 95% CI [-7.74, 0.15] | Lower score suggesting better effect |

CI: confidence interval; GRADE: The Grading of Recommendations Assessment, Development and Evaluation; MD: mean difference; RCT: randomized controlled trial; RR: relative risk; UPDRS: Unified Parkinson’s Disease Rating Scale

Reference

1. Fu B, Lun X, Rong LLin WD, Electroacupuncture at head and du pulse acpoints for treatment of Parkinson disease: Randomized controlled observation. Chinese Journal of Clinical Rehabilitation2004;8:4524-4525.

2. 杨秀毅,电针刺激联合左旋多巴治疗帕金森病的疗效观察. 中国实用神经疾病杂志2016;19:103-105.

3. 王晓平, 罗永杰, 吴景芬, 李晓佳陈凯, 多巴丝肼联合电针对老年帕金森病患者血清白细胞介素-β、胱抑素c及血清离子水平的影响. 中国老年学杂志2016;36:4301-4303.

Supplementary Table 3.4: Summary of findings table: combination treatment with anti-parkinsonianism drugs and scalp acupuncture for idiopathic Parkinson’s disease

| Outcome (clinical importance) | Total number of patients (number of included studies) | Level of evidence (GRADE) | Relative risks (95% CI) | Anticipated absolute effects (95% CI) | | Comments |
| --- | --- | --- | --- | --- | --- | --- |
|  |  |  |  | Controlled group | Intervention group |  |
| Webster scale (critical) | 65 (1 RCTs) | ⊕⊝⊝⊝ Insufficient |  |  | MD -1.97, 95% CI [-3.73, -0.21] | Lower score suggesting better effect |

CI: confidence interval; GRADE: The Grading of Recommendations Assessment, Development and Evaluation; MD: mean difference; RCT: randomized controlled trial; UPDRS: Unified Parkinson’s Disease Rating Scale

Reference

1. Zhang WG, Wang GBQin Y, Scalp acupuncture for treatment of parkinson disease: 32case. Guangming J Chin Med2002;17:55-57.

2. 杨焱陈, 头针治疗帕金森病30例的临床观察. 针灸临床杂志2004;20:36.

3. 刘丹, 刘芳邵滢如, 针刺舞蹈震颤控制区配合药物治疗帕金森病疗效观察. 上海针灸杂志2015;34:825-826.

Supplementary Table 4.1: Summary of findings table: moxibustion for idiopathic Parkinson’s disease

| Outcome (clinical importance) | Total number of patients (number of included studies) | Level of evidence (GRADE) | Relative risks (95% CI) | Anticipated absolute effects (95% CI) | | Comments |
| --- | --- | --- | --- | --- | --- | --- |
|  |  |  |  | Controlled group | Intervention group |  |
| UPDRS total score (critical) | 293 (5) | ⊕⊝⊝⊝  Insufficient^a,b,c^ |  |  | MD -8.75, 95% CI [-12.54, -4.95] | Lower score suggesting better effect |
| UPDRS Ⅲ score (critical) | 293 (5) | ⊕⊕⊝⊝  Low^a,b^ |  |  | MD -1.92, 95% CI [-3, -0.84] | Lower score suggesting better effect |

CI: confidence interval; GRADE: The Grading of Recommendations Assessment, Development and Evaluation; MD: mean difference; RCT: randomized controlled trial; RR: relative risk; UPDRS: Unified Parkinson’s Disease Rating Scale; a: Downgraded once due to unclear risk of bias in participants and outcome assessor blinding in most studies; b: Downgraded due to statistical heterogeneity; c: Downgraded due to small sample size

Reference

1. X. Deng, Clinical Observation on Moxibustion in the treatment of Myotonic myopathies of Parkinson’s Disease, Guangzhou University of Chinese Medicine, 2010.

2. J. Lei, Mild moxibustion with Madopar in Parkinson’s patients to improve the clinical observation of daily activities, Hubei University of Chinese Medicine, 2011.

3. X. Wen, Y.w. Li, and Q. Duan,"Abdominal Acupuncture plus Moxibustion for Treatment of Parkinson Disease Rigidity in 30 Patients," Academic Journal of Guangzhou Medical College, vol. 36, no. 01, pp. 59-61, 2008.

4. J.-m. Zhang, D. HU, and L. XU, "Observations on the Efficacy of Heavy Moxibustion on Pont Yongquan as Main Treatment for Parkinson’s Disease," Shanghai Journal of Acupuncture and Moxibustion, no. 11, 2012.

5. P. Zhang, Clinical study on abdominal-acupuncture binding moxibustion in the treatment of Parkinson’s disease, Guangzhou University of Chinese Medicine,2008.

Supplementary Table 4.2: Summary of findings table: combination treatment with direct moxibustion, acupuncture and anti-parkinsonianism drugs for idiopathic Parkinson’s disease

| Outcome (clinical importance) | Total number of patients (number of included studies) | Level of evidence (GRADE) | Relative risks (95% CI) | Anticipated absolute effects (95% CI) | | Comments |
| --- | --- | --- | --- | --- | --- | --- |
|  |  |  |  | Controlled group | Intervention group |  |
| UPDRS total score (critical) | 60 (1) | ⊕⊕⊝⊝  Low^a,b^ |  |  | MD -7.07, 95% CI [-11.30, -2.84] | Lower score suggesting better effect |

CI: confidence interval; GRADE: The Grading of Recommendations Assessment, Development and Evaluation; MD: mean difference; RCT: randomized controlled trial; UPDRS: Unified Parkinson’s Disease Rating Scale; a: Downgraded once due to unclear risk of bias in participants and outcome assessor blinding in most studies; b: Downgraded due to small sample size

Reference

X. Wen, Y.w. Li, and Q. Duan,"Abdominal Acupuncture plus Moxibustion for Treatment of Parkinson Disease Rigidity in 30 Patients," Academic Journal of Guangzhou Medical College, vol. 36, no. 01, pp. 59-61, 2008.

Supplementary Table 4.3: Summary of findings table: combination treatment with Moxa-stick moxibustion and anti-parkinsonianism drugs for idiopathic Parkinson’s disease

| Outcome (clinical importance) | Total number of patients (number of included studies) | Level of evidence (GRADE) | Relative risks (95% CI) | Anticipated absolute effects (95% CI) | | Comments |
| --- | --- | --- | --- | --- | --- | --- |
|  |  |  |  | Controlled group | Intervention group |  |
| UPDRS total score (critical) | 58 (1) | ⊕⊕⊝⊝  Low^a,b^ |  |  | MD –5.84, 95% CI [-11.64, 0.68] | Lower score suggesting better effect |

CI: confidence interval; GRADE: The Grading of Recommendations Assessment, Development and Evaluation; MD: mean difference; RCT: randomized controlled trial; UPDRS: Unified Parkinson’s Disease Rating Scale; a: Downgraded once due to unclear risk of bias in participants and outcome assessor blinding in most studies; b: Downgraded due to small sample size

Reference

J. Lei, Mild moxibustion with Madopar in Parkinson’s patients to improve the clinical observation of daily activities, Hubei University of Chinese Medicine, 2011.

Supplementary Table 4.4: Summary of findings table: combination treatment with warm-needling acupuncture and swallowing exercises for idiopathic Parkinson’s disease

| Outcome (clinical importance) | Total number of patients (number of included studies) | Level of evidence (GRADE) | Relative risks (95% CI) | Anticipated absolute effects (95% CI) | | Comments |
| --- | --- | --- | --- | --- | --- | --- |
|  |  |  |  | Controlled group | Intervention group |  |
| Total effectiveness rate (critical) | 58 (1 RCT) | ⊕⊝⊝⊝  Insufficient^a^ | RR 1.67, 95% CI [1.11 to 2.50] |  | 335 more per 1000, [55 more, 750 more] | More events suggesting better effects |

CI: confidence interval; GRADE: The Grading of Recommendations Assessment, Development and Evaluation; MD: mean difference; RCT: randomized controlled trial; RR: relative risk; UPDRS: Unified Parkinson’s Disease Rating Scale; a: Downgraded twice due to unclear risk of bias in the sequence generation and allocation concealment domains. In addition, there were also concerns that blinding of participants and personnel was not possible in the nature of the intervention(moxibustion) itself. Incomplete outcome data was observed in the study.

Reference

Y. Zhao, P. Liu, M. Wang, and Y. Yin, "Clinical Observation of Needle Warming Moxibustion Combined with Function Training on Dysphagia of Parkinson’s disease," Chinese Medicine Modern Distance Education of China, no. 07, pp. 61-63,2015.

Supplementary Table 5: Summary of findings table: combination treatment with pharmacoacupuncture and anti-parkinsonianism drugs for idiopathic Parkinson’s disease

| Outcome (clinical importance) | Total number of patients (number of included studies) | Level of evidence (GRADE) | Relative risks (95% CI) | Anticipated absolute effects (95% CI) | | Comments |
| --- | --- | --- | --- | --- | --- | --- |
|  |  |  |  | Controlled group | Intervention group |  |
| Total effectiveness rate (important) | 79 (1 RCT) | ⊕⊕⊝⊝  Low^a,b^ | RR 1.06, 95% CI [0.90, 1.26] |  | 51 more per 1000, [85 less, 220 more] | More events suggesting better effect |

CI: confidence interval; GRADE: The Grading of Recommendations Assessment, Development and Evaluation; RCT: randomized controlled trial; RR: relative risk; a: Downgraded once due to unclear risk of bias in participants and outcome assessor blinding in most studies; b: Downgraded due to small sample size

Reference

刘承浩, 王睿, 金亚蓓, 孙占玲, 周翔, and 何嘉莹, "葛根素穴位注射治疗早中期帕金森病:多中心随机对照试验," 针刺研究, no.01, 2015.

Supplementary Table 6.1: Summary of findings table: combination treatment with Qigong, walking exercise and anti-parkinsonianism drugs for idiopathic Parkinson’s disease

| Outcome (clinical importance) | Total number of patients (number of included studies) | Level of evidence (GRADE) | Relative risks (95% CI) | Anticipated absolute effects (95% CI) | | Comments |
| --- | --- | --- | --- | --- | --- | --- |
|  |  |  |  | Controlled group | Intervention group |  |
| UPDRS Ⅲ score (critical) | 141  (2studies) | ⊕⊕⊝⊝  Low |  |  | MD –4.17, 95% CI [-5.43, -2.92] | Lower score suggesting better effect |
| BBS (important) | 89 (1study) | ⊕⊝⊝⊝  Insufficient |  |  | MD 3.30, 95% CI [2.62, 3.98] | Higher score suggesting better effect |
| Total sleep quality in PDSS-2 (critical) | 89 (1study) | ⊕⊕⊝⊝  Low |  |  | MD -11.47, 95% CI [-15.77, -7.17] | Lower score suggesting better effect |
| Motor Symptoms at Night in PDSS-2 (important) | 89 (1study) | ⊕⊝⊝⊝  Insufficient |  |  | MD -4.63, 95% CI [-6.02, -3.24] | Lower score suggesting better effect |
| PD Symptoms at Night in PDSS-2 (important) | 89 (1study) | ⊕⊝⊝⊝  Insufficient |  |  | MD -3.2, 95% CI [-4.37, -1.83] | Lower score suggesting better effect |
| Disturbed Sleep in PDSS-2 (important) | 89 (1study) | ⊕⊕⊝⊝  Low |  |  | MD -3.44, 95% CI [-5.09, -1.79] | Lower score suggesting better effect |

BBS: Berg Balance Scale; CI: confidence interval; GRADE: The Grading of Recommendations Assessment, Development and Evaluation; MD: mean difference; PDSS: Parkinson’s Disease Sleep Scale; RCT: randomized controlled trial; RR: relative risk; UPDRS: Unified Parkinson’s Disease Rating Scale

Reference

Schmitz-Hübsch T, Pyfer D, Kielwein K, Fimmers R, Klockgether T, Wüllner U: Qigong exercise for the symptoms of Parkinson's disease: A randomized, controlled pilot study. Movement Disorders 2006;21:543-548.

Xiao CM, Zhuang YC: Effect of health baduanjin qigong for mild to moderate Parkinson's disease. Geriatrics and Gerontology International 2015

Supplementary Table 6.2: Summary of findings table: Tai chi for idiopathic Parkinson’s disease

| Outcome (clinical importance) | Total number of patients (number of included studies) | Level of evidence (GRADE) | Relative risks (95% CI) | Anticipated absolute effects (95% CI) | | Comments |
| --- | --- | --- | --- | --- | --- | --- |
|  |  |  |  | Controlled group | Intervention group |  |
| UPDRS Ⅲ score (critical) | 216 (3 RCTs) | ⊕⊕⊝⊝  Low |  |  | MD -3.1, 95% CI [-3.86, -2.34] | Lower score suggesting better effect |
| BBS (important) | 85 (2 RCTs) | ⊕⊕⊕⊝  Moderate |  |  | MD 3.52, 95% CI [1.92, 5.12] | Higher score suggesting better effect |
| Numbers of fallers (important) | 260 (2 RCTs) | ⊕⊕⊕⊝  Moderate | OR 0.39, 95% CI [0.19 to 0.79] | 485 per 1000 (31.5%) | 216 fewer per 1000 [from 58 fewer to 333 fewer] | Fewer events suggesting fewer numbers of fallers |

BBS: Berg Balance Scale; CI: confidence interval; GRADE: The Grading of Recommendations Assessment, Development and Evaluation; MD: mean difference; OR: Odds ratio; RCT: randomized controlled trial; UPDRS: Unified Parkinson’s Disease Rating Scale

Reference

1. Zhu Y, Li JX, 朱毅, 李建兴, 李凝, 金宏柱, 华亮, 董卿, 太极拳对早期帕金森病运动控制的影响, Chinese Journal of Rehabilitation Theory and Practice 2011;17:355-358.

2. 李建兴, 太极拳配合美多巴对帕金森病患者的运动控制作用]. 南京中医药大学 2011

3. Li F, Harmer P, Fitzgerald K, Â Eckstrom E, Â Stock R, Â Galver J: Tai chi and postural stability in patients with parkinson's disease. New England journal of medicine.

4. Li F, Harmer P: Tai chi training to reduce falls in patients with parkinson's disease – a cost-effectiveness analysis. Movement Disorders 2013;28:S104.

5. Li SJ, Mao ZJ, 季苏琼, 毛志娟, 杨清梅, 高红铃, 薛峥, 太极拳锻炼对帕金森患者的疗效观察. Chinese Journal of Rehabilitation 2016;31:51-53.

6. 管细红, 唐霞珠, 刘建民, 太极拳训练对帕金森病病人步行能力及害怕跌倒的影响, Chinese Nursing Research 2016;30:3514-3517.

7. 管细红, 刘芸, 张琼, 杨平, 太极拳训练对帕金森患者心理健康及生活质量的影响, China Journal of Health Psychology 2016;24:1538-1541.

8. Choi HJ, Garber CE, Jun TW, Jin YS, Chung SJ, Kang HJ: Therapeutic effects of tai chi in patients with Parkinson's disease. ISRN Neurology 2013;1

9. Nocera JR, Amano S, Vallabhajosula S, Hass CJ: Tai chi exercise to improve non-motor symptoms of Parkinson's disease. Journal of yoga & physical therapy 2013;3

10. Gao Q, Leung A, Yang Y, Wei Q, Guan M, Jia C: Effects of tai chi on balance and fall prevention in parkinson's disease: A randomized controlled trial. Clinical Rehabilitation. DOI: 10.1177/0269215514521044.

Supplementary Table 7: All recommendations summary

| Clinical questions | Recommendations |
| --- | --- |
| Is concomitant administration of herbal medicines and anti-parkinsonian drug therapy a more effective symptomatic treatment for IPD than monotherapy with anti-parkinsonian agents? | Concomitant treatment with herbal medicines and anti-parkinsonian drugs should be considered in patients with IPD (strength of recommendation: B/level of evidence: low). |
| Does administration of Bosin-yanggan-sigpung-bang herbal medicine with anti-parkinsonian drug therapy improve symptoms more than anti-parkinsonian drugs alone in patients diagnosed with IPD? | Concomitant administration of Bosin-yanggan-sigpung-bang herbal medicine and anti-parkinsonian drug therapy may be considered in patients with IPD (strength of recommendation: C/level of evidence: insufficient). |
| Does administration of Bosin-hwalhyeol-cheobang herbal medicine with anti-parkinsonian drug therapy improve symptoms more than anti-parkinsonian drug therapy alone in patients diagnosed with IPD? | Concomitant administration of Bosin-hwalhyeol-tonglag-cheobang herbal medicine and anti-parkinsonian drug therapy may be considered in patients with IPD (strength of recommendation: C/level of evidence: moderate). |
| Does administration of Sugji-pyeongjeon-tang herbal medicine with anti-parkinsonian drugs improve symptoms more than anti-parkinsonian drug therapy alone in patients diagnosed with IPD? | Concomitant use of Sugji-pyeongjeon-tang herbal medicine and anti-parkinsonian drug therapy should be considered in patients with IPD (strength of recommendation: B/level of evidence: low). |
| Does a combination of anti-parkinsonian drug therapy and acupuncture improve symptoms more than anti-parkinsonian drug therapy alone in patients diagnosed with IPD? | Concomitant use of acupuncture and anti-parkinsonian drug therapy should be considered in patients with IPD (strength of recommendation: B/level of evidence: low).  GB20, LR3, GB34, LI4, GV20, KI39, LI11, GV16, BL10, BL40, GB6, GV1, and PC6 can be considered for use in acupuncture. |
| Does combination treatment with anti-parkinsonian drug therapy and manual acupuncture improve symptoms more than anti-parkinsonian drug therapy alone in patients diagnosed with IPD? | Concomitant use of manual acupuncture and anti-parkinsonian drug therapy should be considered in patients with IPD (strength of recommendation: B/level of evidence: low). |
| Does combination treatment with anti-parkinsonian drug therapy and electroacupuncture improve symptoms more than anti-parkinsonian drug therapy alone in patients diagnosed with IPD? | Concomitant use of electroacupuncture and anti-parkinsonian drug therapy may be considered in patients with IPD (strength of recommendation: C/level of evidence: low).  If the patient’s chief complaints are tremor and rigidity, electroacupuncture should be applied carefully so as not to trigger overstimulation. |
| Does a combination of anti-parkinsonian drug therapy and scalp acupuncture improve symptoms more that anti-parkinsonian drug therapy alone in patients with IPD? | Concomitant use of scale acupuncture and anti-parkinsonian drugs may be considered in patients with IPD (strength of recommendation: good practice point/level of evidence: insufficient). |
| Does moxibustion improve symptoms in patients with IPD? | Moxibustion may be considered in patients with IPD (strength of recommendation: C/level of evidence: low).  Care should be taken not to cause adverse events by excessive stimulation if the main symptoms are tremor and rigidity. |
| Does combination treatment with direct moxibustion, acupuncture, and anti-parkinsonian drug therapy improve symptoms in patients diagnosed with IPD? | A combination of direct moxibustion, acupuncture, and anti-parkinsonian drug therapy may be considered in patients with IPD (strength of recommendation: C/level of evidence: low). Care should be taken not to cause adverse events by excessive stimulation if the patient‘s main symptoms are tremor and rigidity. |
| Does a combination of Moxa-stick moxibustion and anti-parkinsonian drug therapy improve symptom in patients diagnosed with IPD? | Concomitant treatment with Moxa-stick moxibustion and anti-parkinsonian drug therapy may be considered in patients with IPD (strength of recommendation: C/level of evidence: low).  Care should be taken not to cause adverse events by excessive stimulation if the patient’s main symptoms are tremor and rigidity. |
| Does combination treatment with warm-needling acupuncture and swallowing exercises improve dysphagia symptoms in patients diagnosed with IPD? | Combination treatment with warm-needling acupuncture and swallowing exercises may be considered for the treatment of dysphagia in patients with IPD (strength of recommendation: C/level of evidence: low). |
| Does a combination of bee venom acupuncture and anti-parkinsonian drug therapy improve symptoms in patients diagnosed with IPD? | Concomitant treatment with bee venom acupuncture and anti-parkinsonian drug therapy is recommended in patients with IPD (strength of recommendation: GPP/level of evidence: insufficient). |
| Does a combination of pharmaco-acupuncture and anti-parkinsonian drug therapy improve symptoms in patients diagnosed with IPD? | Concomitant treatment with pharmaco-acupuncture and anti-parkinsonian drug therapy is recommended in patients with IPD (strength of evidence: GPP/level of evidence: insufficient). |
| Does a combination of Qigong, walking exercise, and anti-parkinsonian drug therapy improve motor function and sleep quality in patients diagnosed with IPD? | Concomitant treatment with qigong, walking exercise, and anti-parkinsonian drug therapy should be considered in patients with IPD (strength of recommendation: B/level of evidence: low). |
| Does Tai chi improve motor function in patients diagnosed with IPD? | Tai chi can be considered in patients with IPD (strength of recommendation: B/level of evidence: low). |
